# Supplementary material for: Ancient genomes from the Himalayas illuminate the genetic history of Tibetans and their Tibeto-Burman speaking neighbors
Source: Nat Commun. 2022 Mar 8;13:1203. doi: 10.1038/s41467-022-28827-2 (PMC8904508; doi:10.1038/s41467-022-28827-2)
Supplement: Supplementary file 1 — Supplementary Information [file 41467_2022_28827_MOESM1_ESM.pdf]

Supplementary Information

Ancient genomes from the Himalayas illuminate the genetic history of Tibetans and their Tibeto-Burman speaking neighbors

Chi-Chun Liu, David Witonsky, Anna Gosling, Juhyun Lee, Harald Ringbauer, Richard Hagan, Nisha Patel, Raphaela Stahl, John Novembre, Mark Aldenderfer*, Christina Warinner*, Anna Di Rienzo*, Choongwon Jeong*

* Correspondence to: maldenderfer@ucmerced.edu (M.A.), warinner@fas.harvard.edu (C.W.), dirienzo@bsd.uchicago.edu (A.D.), cwjeong@snu.ac.kr (C.J.)

This file includes:

Supplementary Text 1
Supplementary Figures 1 to 14
Supplementary Tables 1 to 10

Supplementary Text 1. Regional and archaeological context of the samples

The archaeological sites from which the samples were recovered are found in the newly designated province of Gandaki Pradesh, which is located in north-central Nepal and borders the Tibet Autonomous Region. Five of the six sites—Chokhopani, Lubrak, Rhirhi, Mebrak, and Samdzong—are in the Mustang District, while Kyang is in the Manang District, which lies to the east of Mustang (Fig. 1).

Mustang lies within a rain shadow created by the Annapurna and Dhaulagiri massifs which limit the amount and frequency of rainfall during the summer monsoon and snowfall in the winter. Annual precipitation and temperature vary within the region according to elevation. Mustang's capital, Jomsom, is located at 2,729 masl and has an annual precipitation of 307 mm and an average annual temperature of 10.9˚C. With each 1,000 m rise in elevation, the average annual temperature drops by 6˚C, and annual precipitation throughout the trans-Himalayan region of Mustang is less than 200 mm[1]. These conditions have promoted the excellent preservation of organic and metallic cultural materials. The primary drainage of Mustang is the Kali Gandaki river, which has created a natural north-south corridor that ultimately connects South Asia with the Tibetan Plateau through a relatively low pass over the Himalayas (Kora La; 4660 masl). This corridor promoted extensive trade and communication between Tibet and South Asia throughout historic periods and undoubtedly into the prehistoric past.

Manang has a similar climate to that of Mustang but is not fully within the rain shadow defined by the Annapurna and Dhaulagiri massifs. Nevertheless, the relatively arid and cold climate also promotes the preservation of organic and metallic cultural materials. The primary drainage is the Nar-Phu khola which runs north-south from its origins in the Himalayas to the Marsyandi river, Unlike Mustang, however, there is no easily accessible north-south route from Manang across the Himalayas to the Tibetan Plateau. An east-west route across the Thorung La pass (5416 masl) does connect the Nar-Phu khola with the Kali Gandaki drainage, thus affording access to the Plateau.

All radiocarbon dates have been calibrated with Calib 8.2/IntCal 20 using the Northern Hemisphere calibration curve and reported at 2 sigma[2]. For those sites with multiple reported dates, all dates were recalibrated and the range of the calibrated dates is reported at 2 sigma. Details of laboratory protocols for radiocarbon dating facilities are described in Supplementary Data 3.

Suila (Soya La; ca. 3900 masl 3140±20; UCIAMS 228061; 1491-1317 BCE): This site is found on the high tablelands approximately 4 km west of the channel of the Kali Gandaki river. It was discovered during road construction in the summer of 2018. The original context, now destroyed, was a small cave just below a high pass that leads to Lo Manthang to the north. Workers recovered human remains and artifacts which were taken to the school in the village of Ghiling which lies just to the south of the pass. Human remains include three crania, a single mandible, and two pairs of femora and tibia. A single, near-complete ceramic vessel with a strap handle was recovered; the vessel fabric is a coarse grey with small inclusions. No other artifacts were recovered.

Lubrak (3000 masl; 2980±20 BP; UCIAMS 204452; 1263-1127 BCE): This site, consisting of two slab-cist chambers, was discovered eroding out of the north bank of the Pandak khola near the modern village of Lubrak. The remaining stratigraphy of the profile suggests that the chambers were placed into specially excavated pits and were then covered over. Each chamber had a single primary inhumation. Unfortunately, many of the skeletal elements of the burials were lost consequent to the erosion of the bank. Artifacts found in the chambers include a large number of ceramic vessels, carnelian, glass, shell and possibly metal beads, and copper rings and bangles.

Rhirhi (3200 masl; multiple dates with a range of 805-767 BCE; Supplementary Data 3): Rhirhi is located on the western bank of the Kali Gandaki on a plateau that overlooks the river below. The cliff face is

dotted with caves, some natural and others excavated by humans. A large looter's pit was excavated at some time in the past and remains of five individuals and artifacts were found scattered upon the surface. Fragments of ceramics similar to those found at Chokhopani and Lubrak were discovered. It is likely that the original burial context was a pit similar to those at Chokhopani. Radiocarbon dating was done on two of the individuals from the site. The site is archaeologically contemporary with Chokhopani.

Chokhopani (2800 masl; 2575±19 BP; Hd 15597-15059; 801-770 BCE): The site is found on the south side of the Kali Gandaki river across from the modern village of Marpha[3]. The site, entirely destroyed by the installation of a micro-hydro facility and subsequent looting, consisted of at least three burial chambers found in human-made caves which contained at least 21 individuals. A communal burial facility, interments were placed in pits excavated into the floors of the caves, which themselves were created by ancient peoples. Cultural materials present included wooden vessels, birch bark containers, carnelian and faience beads, copper bracelets, and ceramics[4]. Large copper sheets that resemble processed animal hides were also recovered; these suggest trade or exchange relationships with South Asia. Some of the artifacts, including human remains, were recovered by the Department of Archaeology, and placed in the Kapilvastu Museum in southern Nepal. The teeth examined in this manuscript were obtained from that facility.

Mebrak (3600 masl; multiple dates with a range of c. 500 BCE- CE 1; Supplementary Data 3) Mebrak, more correctly identified as Mebrak 63[5,6] is found in the Dzong khola, a tributary of the Kali Gandaki, near the modern village of Jharkot. The site is one of a series of caves excavated in a sheer cliff face some 30m above the ground surface. It is a communal tomb used sporadically over the dated range of the site and contains at least 27 individuals. Some individuals were partially mummified by the aridity of the climate. An important feature of mortuary practice was the bed coffin; the dead were placed carefully upon them but were pushed aside when newly deceased individuals were placed in the cave. The preservation of organic materials is extraordinary and includes a wide range of wooden, bamboo, textiles, leather, and other materials. Glass and carnelian beads are abundant. Metal artifacts, however, are not common. The site is archaeologically contemporary with Kyang, which is a two-day trip to the east across the Thorung La pass.

Kyang (3900 masl; multiple dates with a range of 695-206 BCE with the majority of the dates falling within the range of 399-199 BCE; Supplementary Data 3): Kyang is found in Manang district on the east side of the Nar-Phu khola. The site is formed by a natural crevice in the steep face of a cliff and is at least 50m above the local ground surface. The crevice is fronted by architectural remains of walls, platforms, and other features. A staircase, built into the crevice, affords access into the chamber. The site has been looted and only a small number of wooden, bamboo, and other organic materials remain. A collective burial, at least 23 individuals were interred at the site over the range of its use[7]. The site is archaeologically contemporary with Mebrak, which lies to the west across the Thorung La pass.

Samdzong (4000 masl; multiple dates with a range of CE 450-650; Supplementary Data 3). Samdzong is located on the east side of the Samdzong khola, which flows into the Kali Gandaki river. The site is found along the sheer face of a west-facing bluff. A seismic event in 2009 collapsed the face of the cliff and exposed ten chambers which were determined to be shaft tombs. A total of 105 individuals were recovered from these collective tombs[6]. Although the context of the tombs was badly disturbed by the seismic event, it appears that the dead were placed upon low bed-like platforms or in some instances, directly upon the surface of the chamber. Although most of the tombs contained quotidian artifacts, such as wooden trays, some ceramics, iron plates and arrowheads, and considerable amounts of horse tack, one tomb—Samdzong 5—contained copper cooking vessels and a cauldron, iron daggers, numerous glass beads, preserved textiles and other decorative objects. Three gold masks were also recovered; these have strong similarities to masks found in western Tibet and northwestern India[8].

In 2016, whole genomes were published for eight ancient individuals from the three type sites (Chokhopani, n=1; Mebrak, n=3; Samdzong, n=4), providing a basic overview of the population history of the region[9].

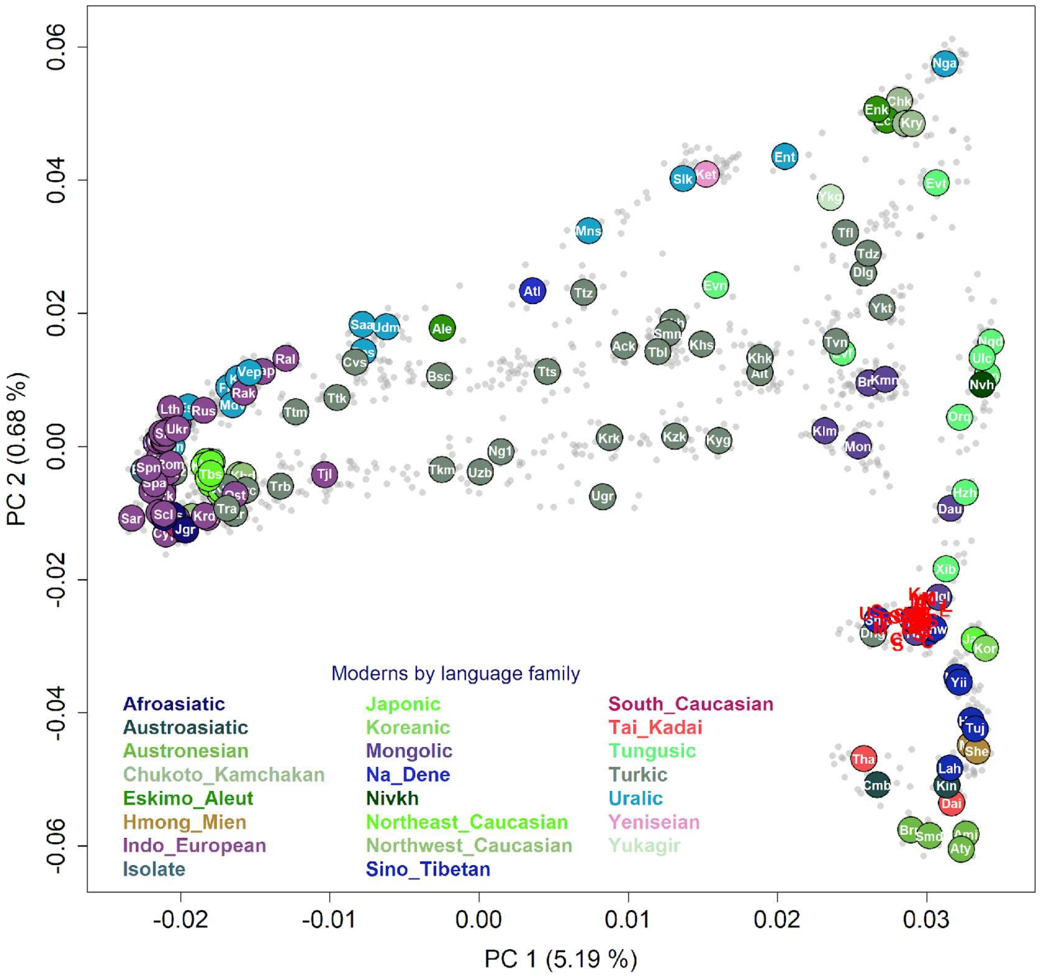

Abz Abazin; Abk Abkhasian; Adg Adygei; Abn Albanian; Ale Aleut; Atl Aleut_Tlingit; Alt Altaian; Ack Altaian_Chelkans; Ami Ami; Arm Armenian; Ahm Armenian_Hemsheni; Aty Atayal; Avr Avar; Azr Azeri; Blk Balkar; Bsc Bashkir; Bsq Basque; Blr Belarusian; Bes Besermyan; Brn Borneo; Blg Bulgarian; Bry Buryat; Cmb Cambodian; Cch Chechen; Chk Chukchi; Cvs Chuvash; Ccs Circassian; Crt Croatian; Cyp Cypriot; Cze Czech; Dai Dai; Drg Darginian; Dau Daur; Dlg Dolgan; Dng Dungan; Ent Enets; Eng English; Ecs Eskimo_CS; Enk Eskimo_NK; Est Estonian; Evn Even; Evf Evenk_FarEast; Evt Evenk_Transbaikal; Ezd Ezid; Fin Finnish; Fre French; Ggz Gagauz; Grg Georgian; Ger German; Grk Greek; Han Han; Hzh Hezhen; Hun Hungarian; Ice Icelandic; Igs Ingushian; Iri Irish; Iru Irish_Ulster; Itn Italian_North; Its Italian_South; Ite Itelmen; Jap Japanese; Jas Jew_Ashkenazi; Jgr Jew_Georgian; Kbd Kabardinian; Ktg Kaitag; Klm Kalmyk; Krc Karachai; Krk Karakalpak; Krl Karelian; Kzk Kazakh; Ket Ket; Khs Khakass; Khk Khakass_Kachins; Kmn Khamnegan; Kin Kinh; Kor Korean; Kry Koryak; Kbc Kubachinian; Kmk Kumyk; Krd Kurd; Kyg Kyrgyz; Lah Lahu; Lak Lak; Lzg Lezgin; Lth Lithuanian; Mlt Maltese; Mns Mansi; Mia Miao; Mld Moldavian; Mon Mongol; Mgl Mongola; Mdv Mordovian; Nan Nanai; Nax Naxi; Ngd Negidal; Nga Nganasan; Nvh Nivh; Ng1 Nogai; Nwg Norwegian; Orc Orcadian; Orq Oroqen; Ost Ossetian; Pol Polish; Rom Romanian; Rus Russian; Rak Russian_Krasnoborsky; Ral Russian_Leshukonsky; Rap Russian_Pinezhsky; Saa Saami; Sar Sardinian; Sct Scottish; Slk Selkup; Smd Semende; She She; Shp Sherpa_Khumbu; Shn Sherpa_Nakatsuka; Shw Sherpa_Wang; Stl Shetlandic; Skh Shor_Khakassia; Smn Shor_Mountain; Scl Sicilian; Srb Sorb; Spa Spanish; Spn Spanish_North; Tbs Tabasaran; Tjl Tajik; Ttk Tatar_Kazan; Ttm Tatar_Mishar; Tts Tatar_Siberian; Ttz Tatar_Zabolotniye; Tha Thai; Tdz Todzin; Tfl Tofalar; Tuu Tu; Tbl Tubalar; Tuj Tujia; Tra Turkish; Trb Turkish_Balikesir; Tkm Turkmen; Tvn Tuvinian; Udm Udmurt; Ukr Ukrainian; Ulc Ulchi; Upm Upper Mustang; Ugr Uygur; Uzb Uzbek; Vep Veps; Xib Xibo; Ykt Yakut; Yii Yi; Ykg Yukagir

Supplementary Fig. 1. aMMD individuals on the top two PCs of present-day Eurasian individuals. We calculated PCs from 2,096 present-day Eurasian individuals in the HO data set and projected aMMD individuals on top of the top PCs. Grey dots represent present-day individuals we used to calculate PCs. Circles represent median positions of present-day groups colored by their language families along with their respective group abbreviations. Red capital letters "U, L, C, R, K, M, S" represent projected aMMD individuals from Suila, Lubrak, Chokhopani, Rhirhi, Kyang, Mebrak, and Samdzong, respectively.

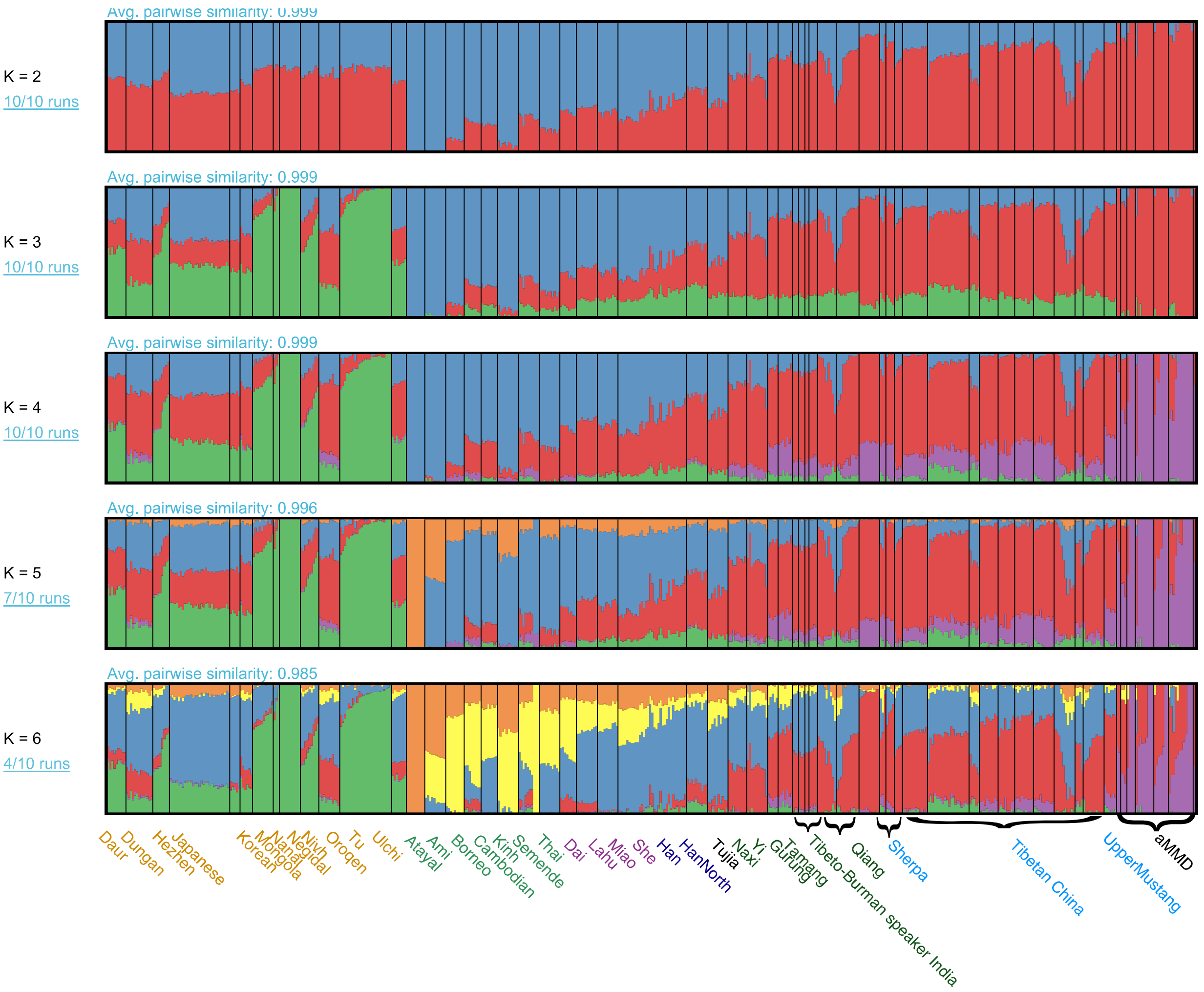

Supplementary Fig. 2. ADMIXTURE plot for East Asian and aMMD individuals. We report the ancestry profiles for the East Asian and aMMD individuals in the HO dataset. We collapse Chakehshanega, Nagaseema, Poumainaga, Nyshi into Tibeto-Burman speaker India; Qiang Danba and Qiang Daufu into Qiang, Sherpa Khumbu, Sherpa_Nakatuska, Sherpa Wang into Sherpa; Tibetan in Yunnan, Chamdo, Gangcha, Gannan, Lhasa, Nagqu, Shannan, Shigatse, Xinlong, Xuhua, Yajiang into Tibetan China; Lubrak, Chokhopani, Rhihi, Mebra, Kyang, Samdzong, Suila into aMMD.

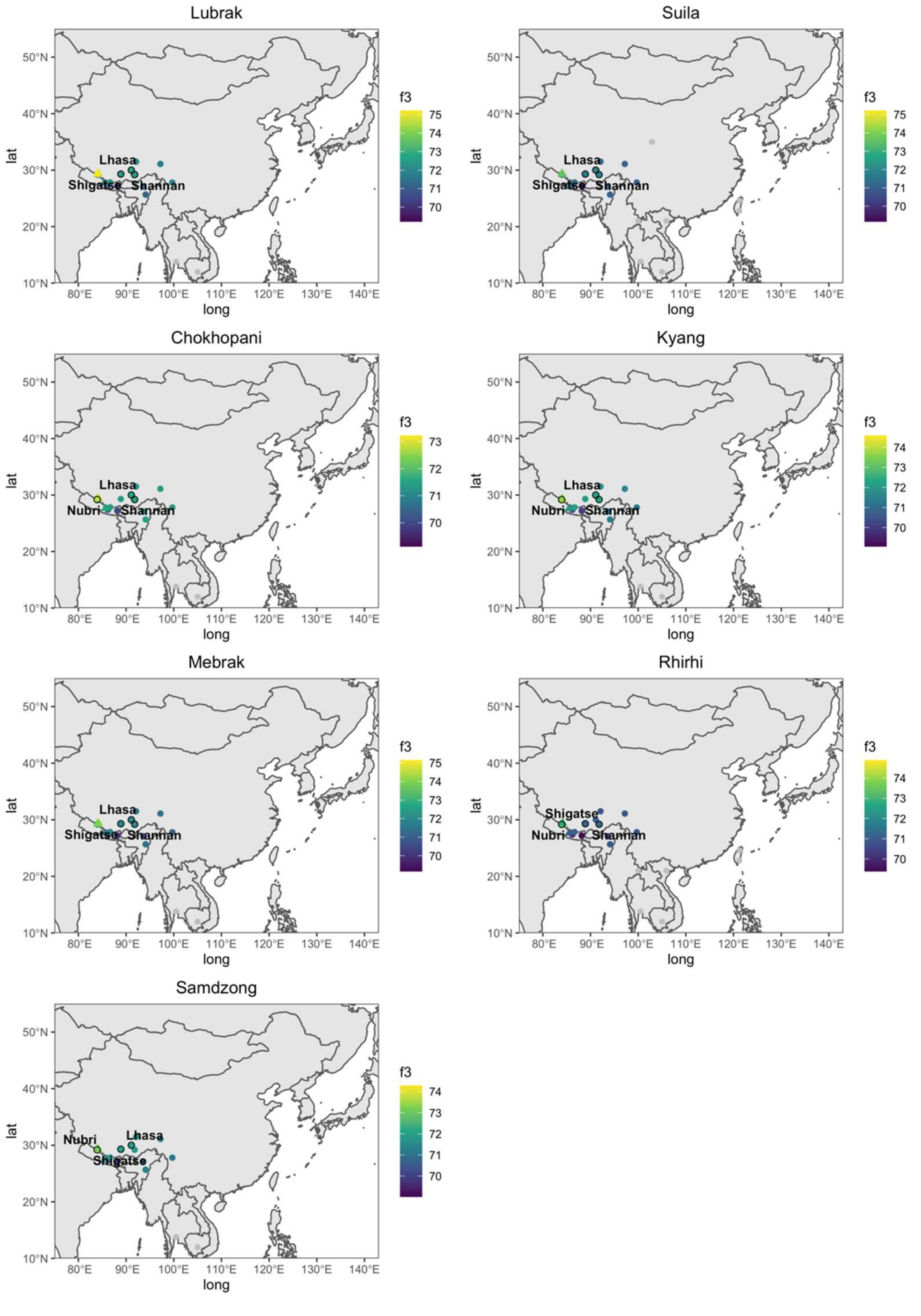

Supplementary Fig. 3. Heat map for outgroup-f_3 statistics showing genetic affinity between aMMD groups and present-day Asian groups in the region (HO dataset). Colors represent f_3(Mbuti; X, aMMD), where X's are present-day groups (circles) and aMMD groups (triangles). We provide the name of the top three signals from present-day populations and annotate them with black borders. Signals lower than 69 are greyed out. The base map was created in R v4.0.5 using publicly available map information from the sf v0.9-8 and rnathralearth v0.2.0 packages.

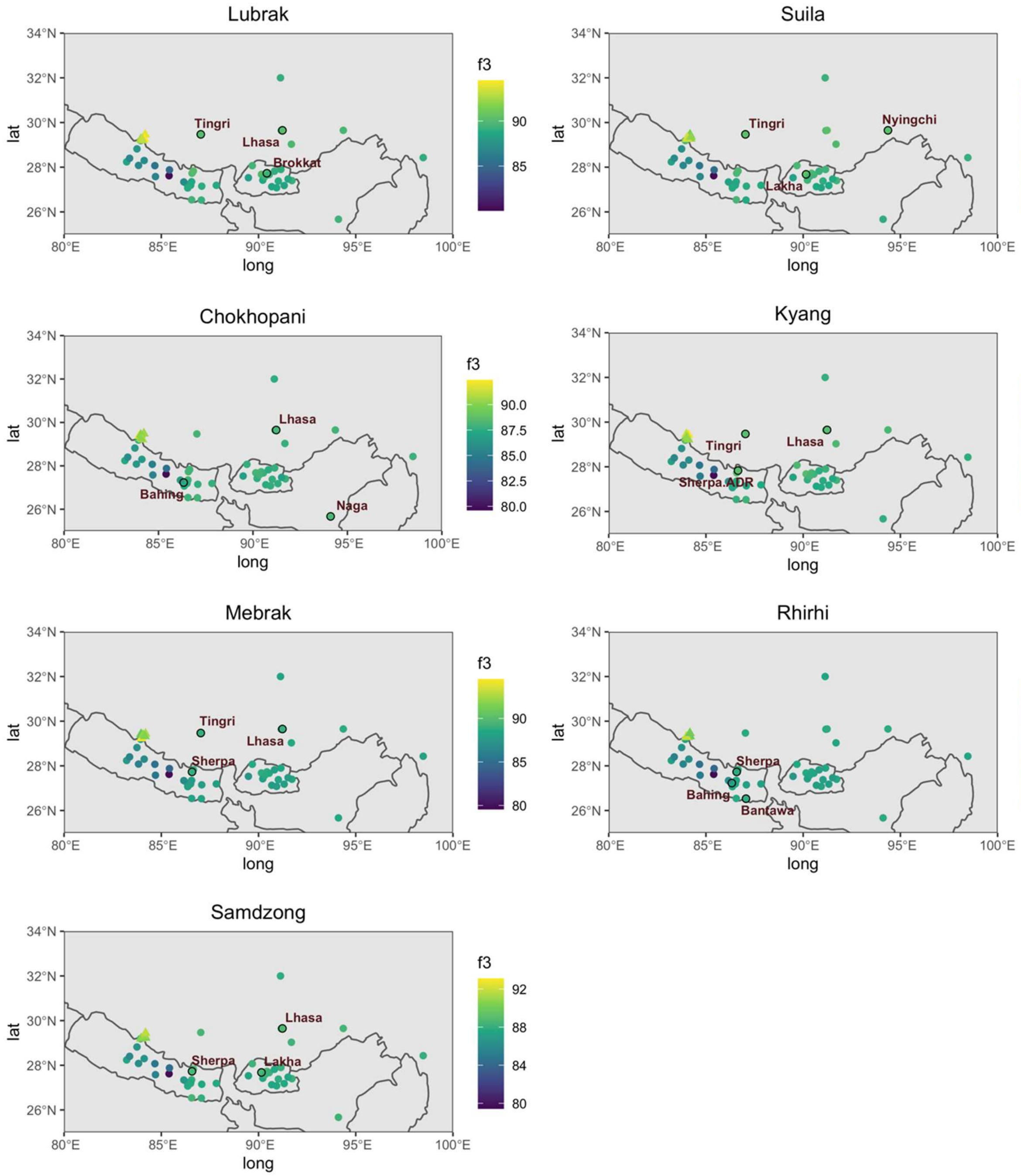

Supplementary Fig. 4. Heat map for outgroup-f_3 statistics showing genetic affinity between aMMD groups and present-day Tibeto-Burman speaking groups in surrounding regions (Illumina dataset). Colors represent f_3(Mbuti; X, aMMD), where X's are present-day groups (circles) and aMMD groups (triangles). We provide the name of the top three signals from present-day populations and annotate them with black borders. The base map was created in R v4.0.5 using publicly available map information from the sf v0.9-8 and rnathralearth v0.2.0 packages.

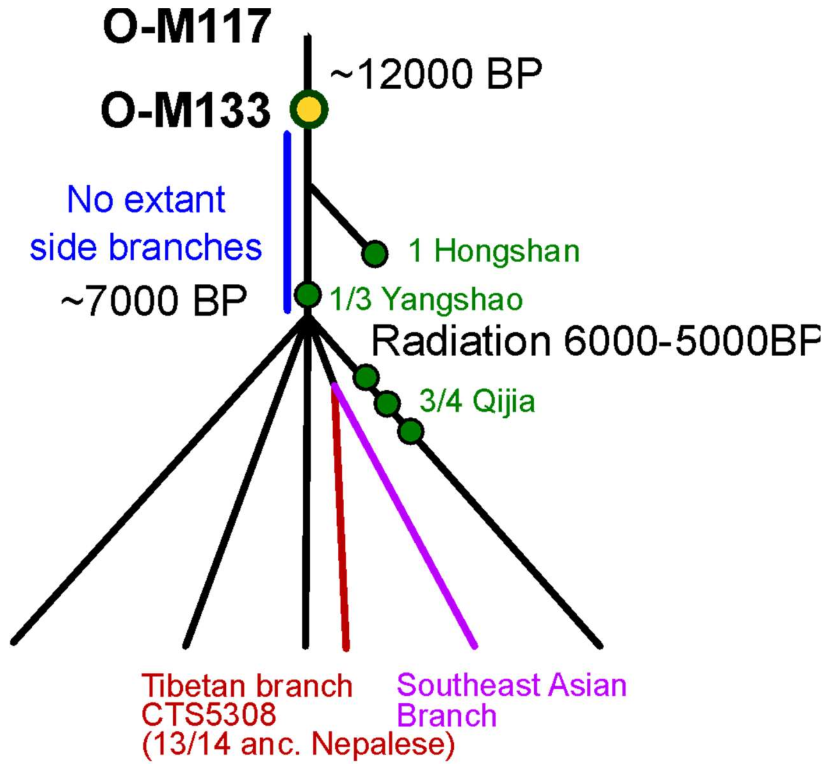

Supplementary Fig. 5. The relationship among Y haplogroup O samples within the aMMD individuals and their lowland neighbors. All aMMD individuals bearing the Y haplogroup O belong to a specific lineage tagged by CTS5308 (marked by brown color, "Tibetan branch CTS5308"). This branch is closest with those commonly found among present-day southeast Asians (marked by purple color, "Southeast Asian branch"). Ancient individuals from the Late Neolithic Qijia culture (ca. 2050-1850 BCE) in the neighboring lowland of the northeastern margin of the Plateau, have Y haplogroup O harboring derived mutations not shared with the aMMD individuals ("3/4 Qijia", marked by green circles). Early barley farmers from the northeastern margin of the Plateau, who post-date the Qijia culture by a few centuries, are thus unlikely a direct source who brought the Y haplogroup O to the ancestors of the aMMD individuals.

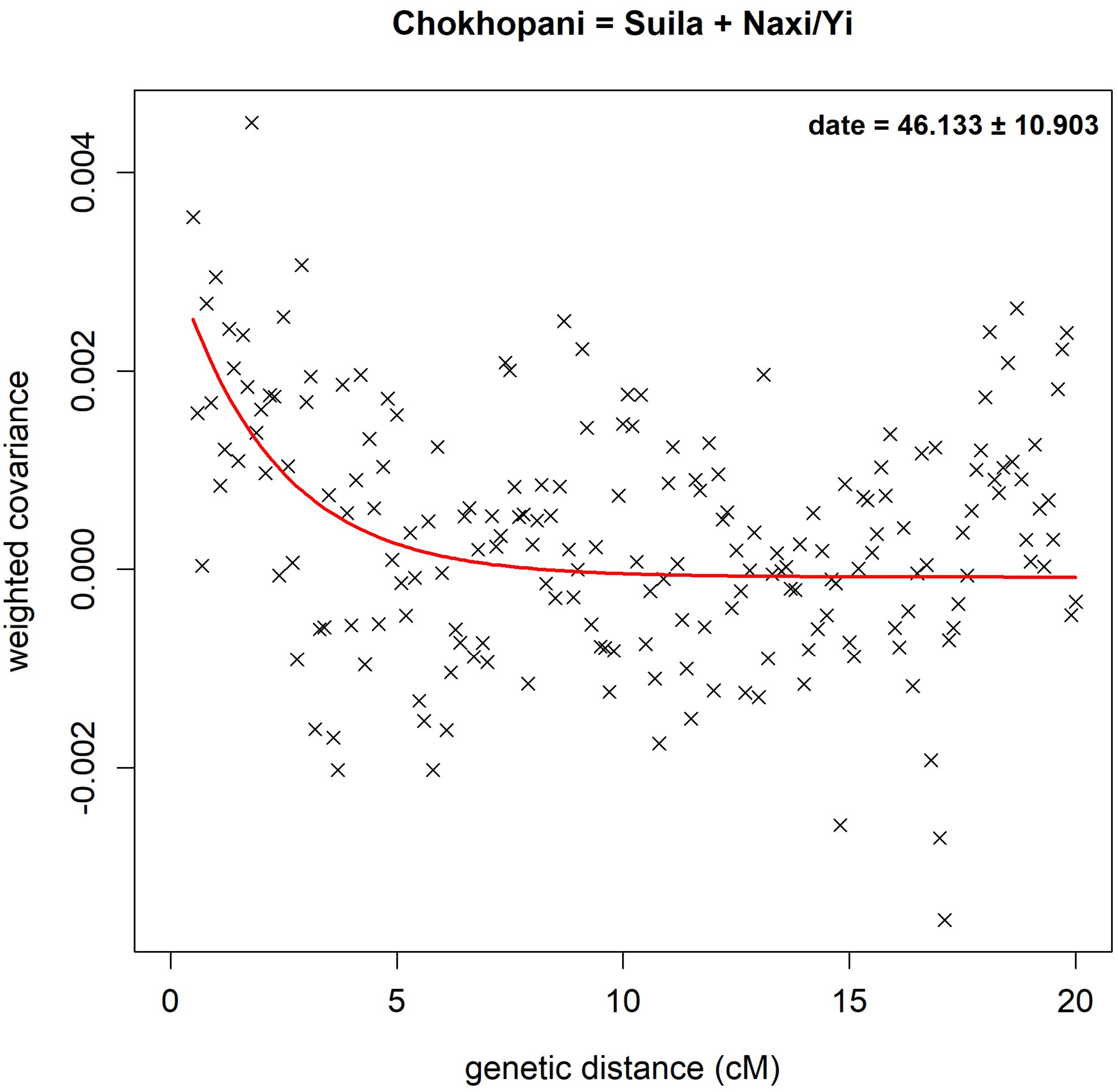

Supplementary Fig. 6. Admixture dating of Chokhopani using DATES. We model Chokhopani using Suila and Naxi+Yi as two sources. DATES models genotype data of each target individual as a linear combination of allele frequency of the two sources and calculates the weighted covariance of the genotype residuals between SNPs by genetic distance. The admixture date estimate and its leave-one-chromosome-out jackknifing standard error are provided in the plot. Chokhopani shows a statistically significant decay.

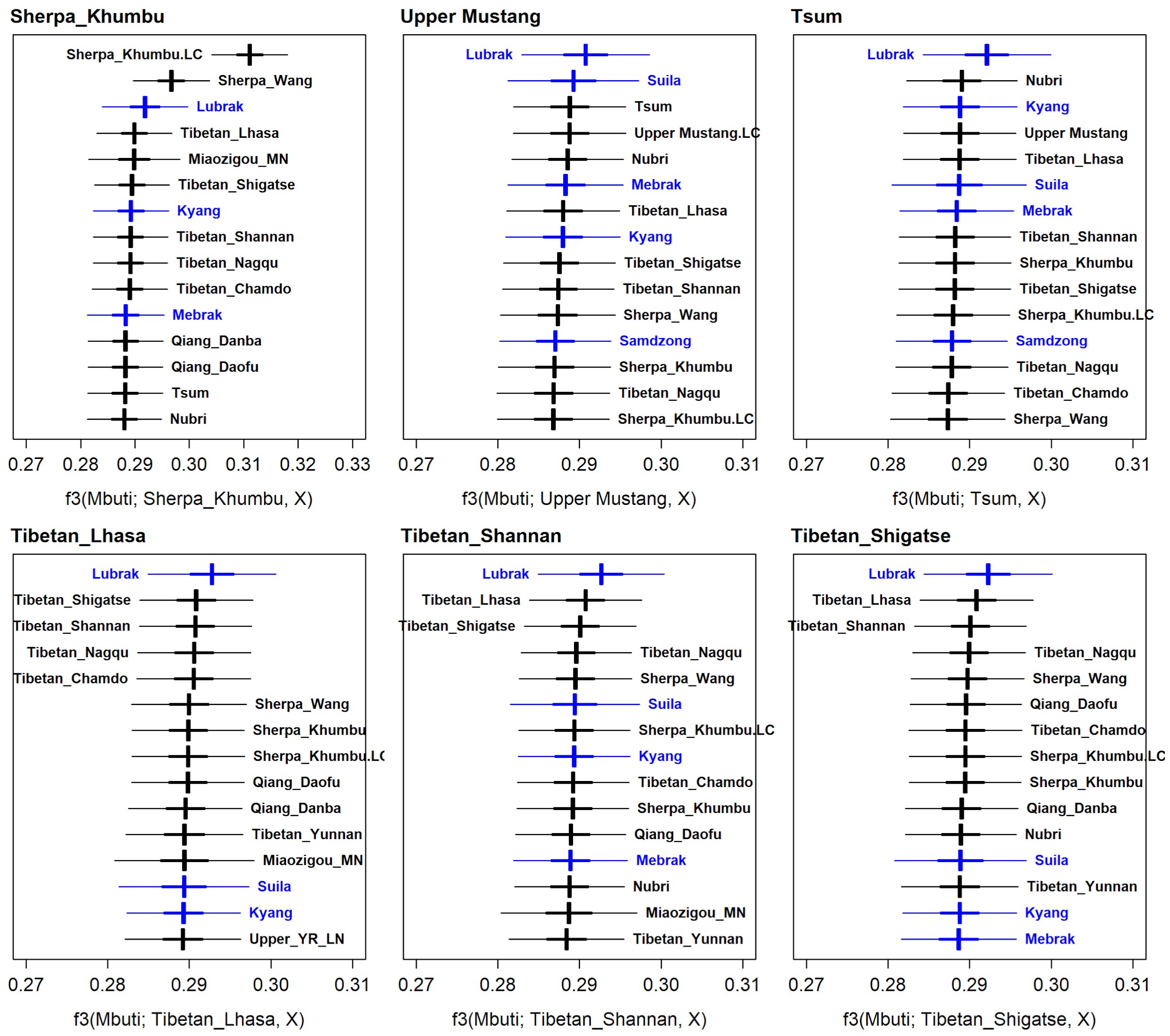

Supplementary Fig. 7. Genetic affinity between present-day Sherpa/Tibetans and ancient/present-day East Asian groups using outgroup-f_3 statistics. Bar plots summarize genetic affinity, measured by f_3(Mbuti; Sherpa/Tibetan, East Asian). The highest 15 signals are presented for each Sherpa/Tibetan group. Vertical bars mark the point estimate of outgroup-f_3 statistic. Thick and thin horizontal bars representing ±1 and 3 standard error measures (SEM) estimated by 5 cM block jackknifing, respectively.

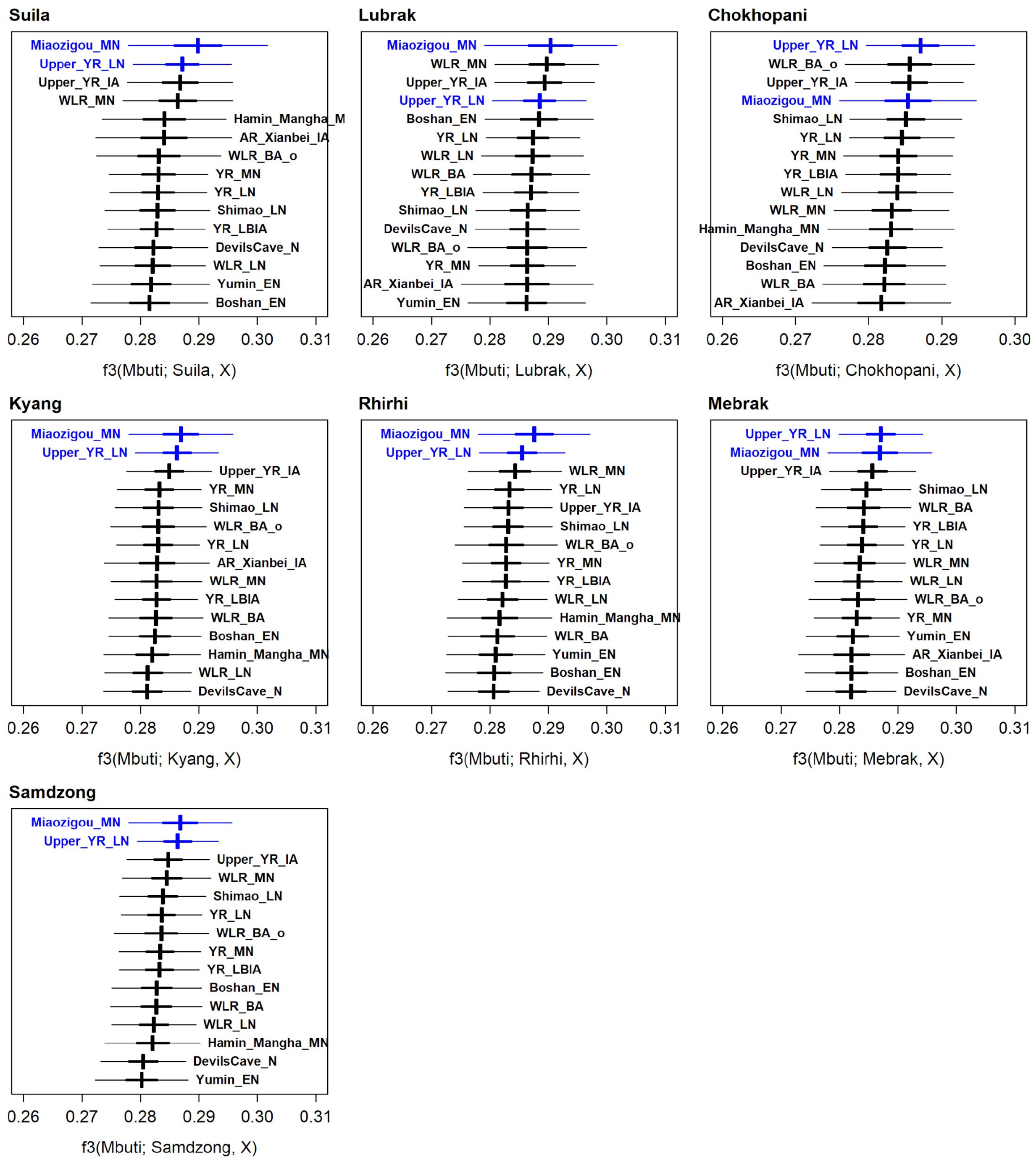

Supplementary Fig. 8. Genetic affinity between aMMDs and ancient lowland East Asian groups using outgroup-f_3 statistics. Bar plots summarize genetic affinity, measured by f_3(Mbuti; ancient East Asian, aMMD). Upper_YR_LN and Miaozigou_MN show the greatest affinity to the aMMD groups. The highest 15 signals are presented for each aMMD group. Vertical bars mark the point estimate of outgroup-f_3 statistic. Thick and thin horizontal bars representing ±1 and 3 SEM estimated by 5 cM block jackknifing, respectively.

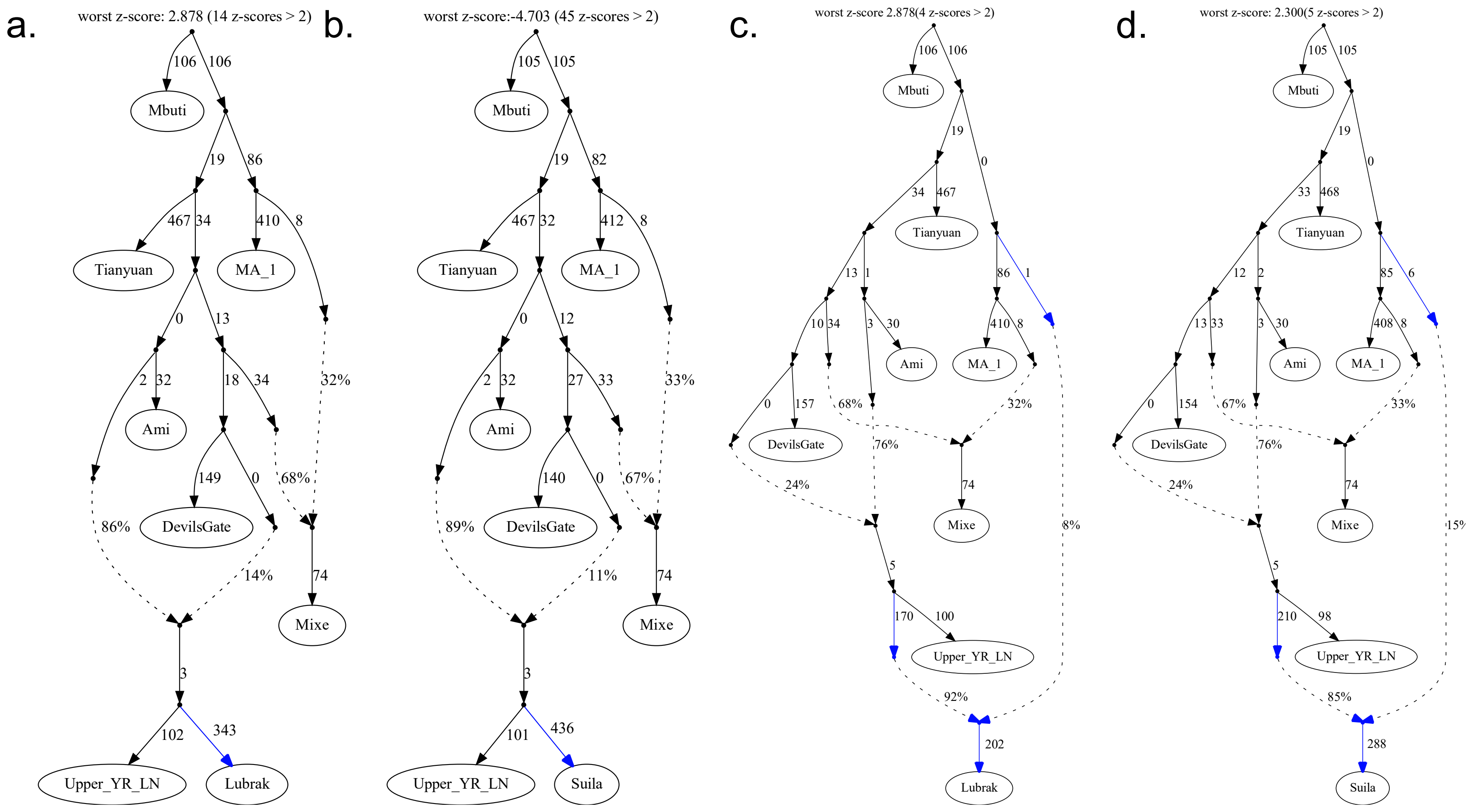

Supplementary Fig. 9. Admixture graph modeling for aMMD groups using qpGraph. (a-b) Modeling aMMD groups as sister groups to Upper_YR_LN results to a large number of significant z-scores. (c-f) Modeling aMMD groups as two-way mixtures with Upper_YR_LN as one source and a deep lineage as the other source. Placing the deep lineage to either the western (c-d) or the eastern (e-f) Eurasian lineage provides an equal fit, therefore we cannot further specify its phylogenetic position in the graph (worst z-score < 3 for all aMMD groups).

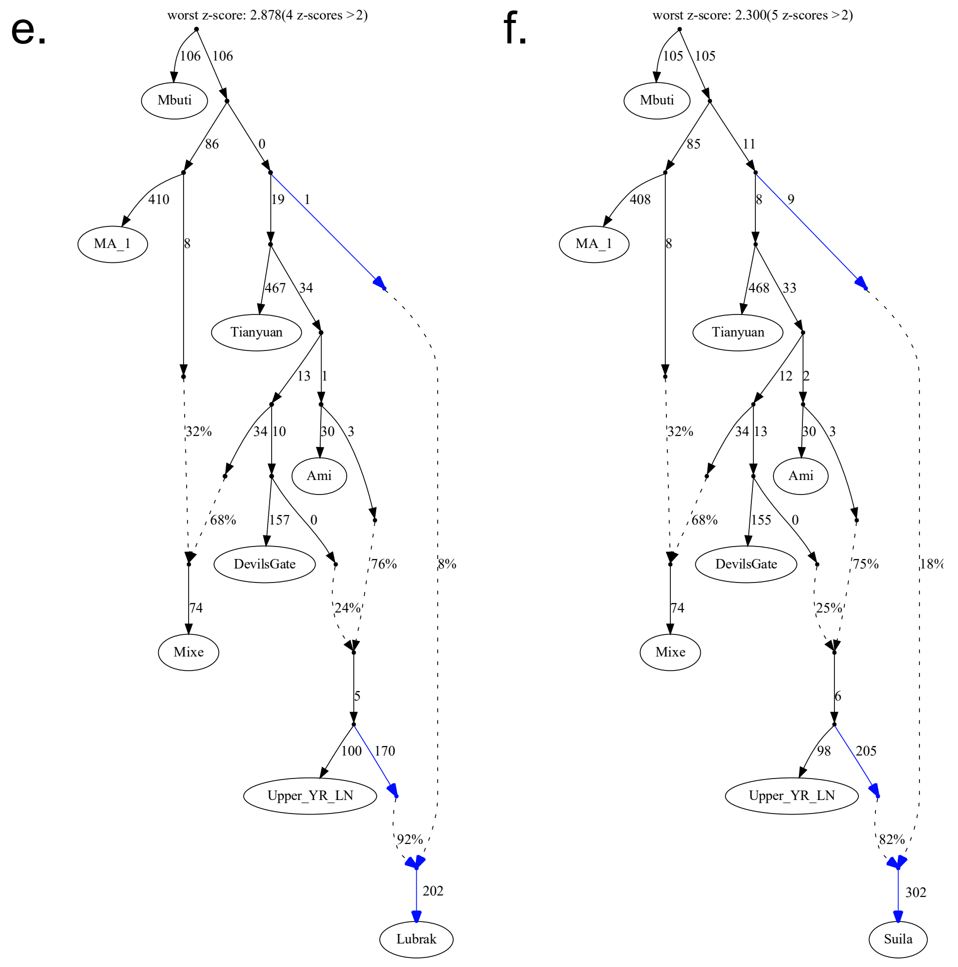

Supplementary Fig. 9. Admixture graph modeling for aMMD groups using qpGraph. (*Continued*)

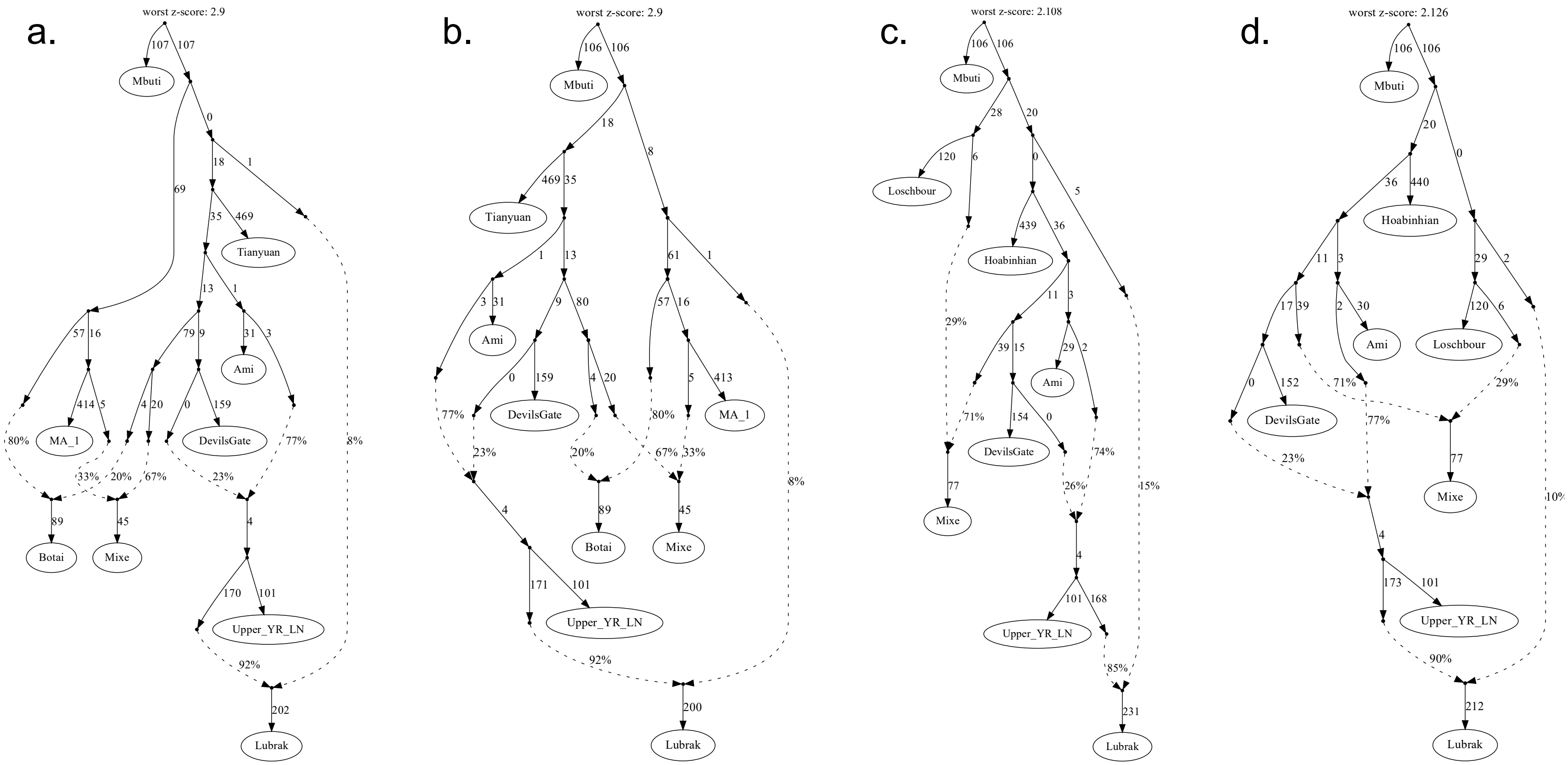

Supplementary Fig. 10. Alternative admixture graph topologies supporting the deep Eurasian ancestry. Several ancient groups including (a-b) Botai, (c-d) Hoabinhian, (e-f) archaic Hominin, and (g-h) Ust'-Ishim are added into the models. The topologies are chosen to be comparable to those in Supplementary Fig. 7, with an emphasis that these topologies provide reasonable fit and these newly added ancient/archaic groups thus cannot tease out the deep ancestry.

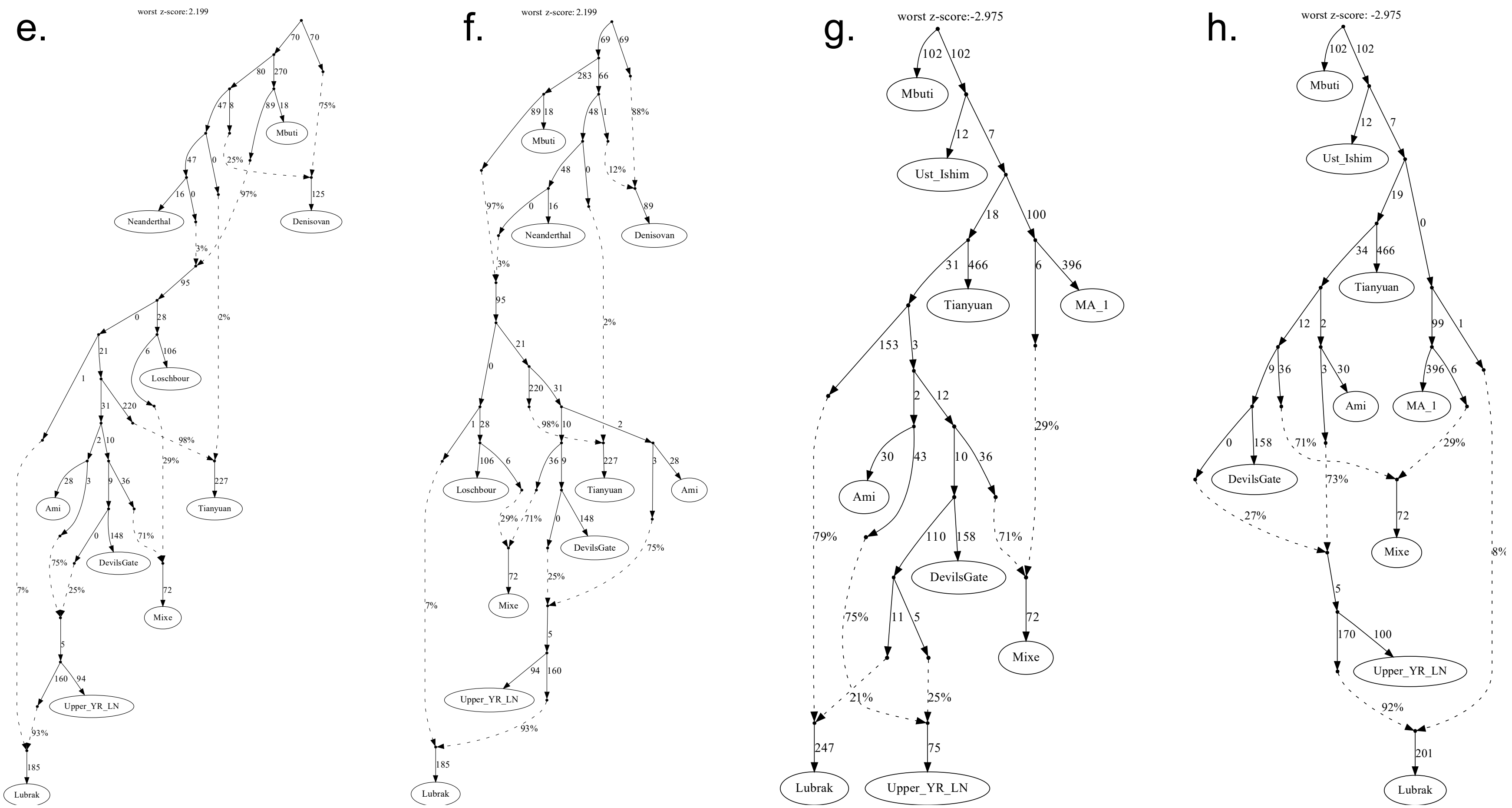

Supplementary Fig. 10. Alternative admixture graph topologies supporting the deep Eurasian ancestry. (*Continued*)

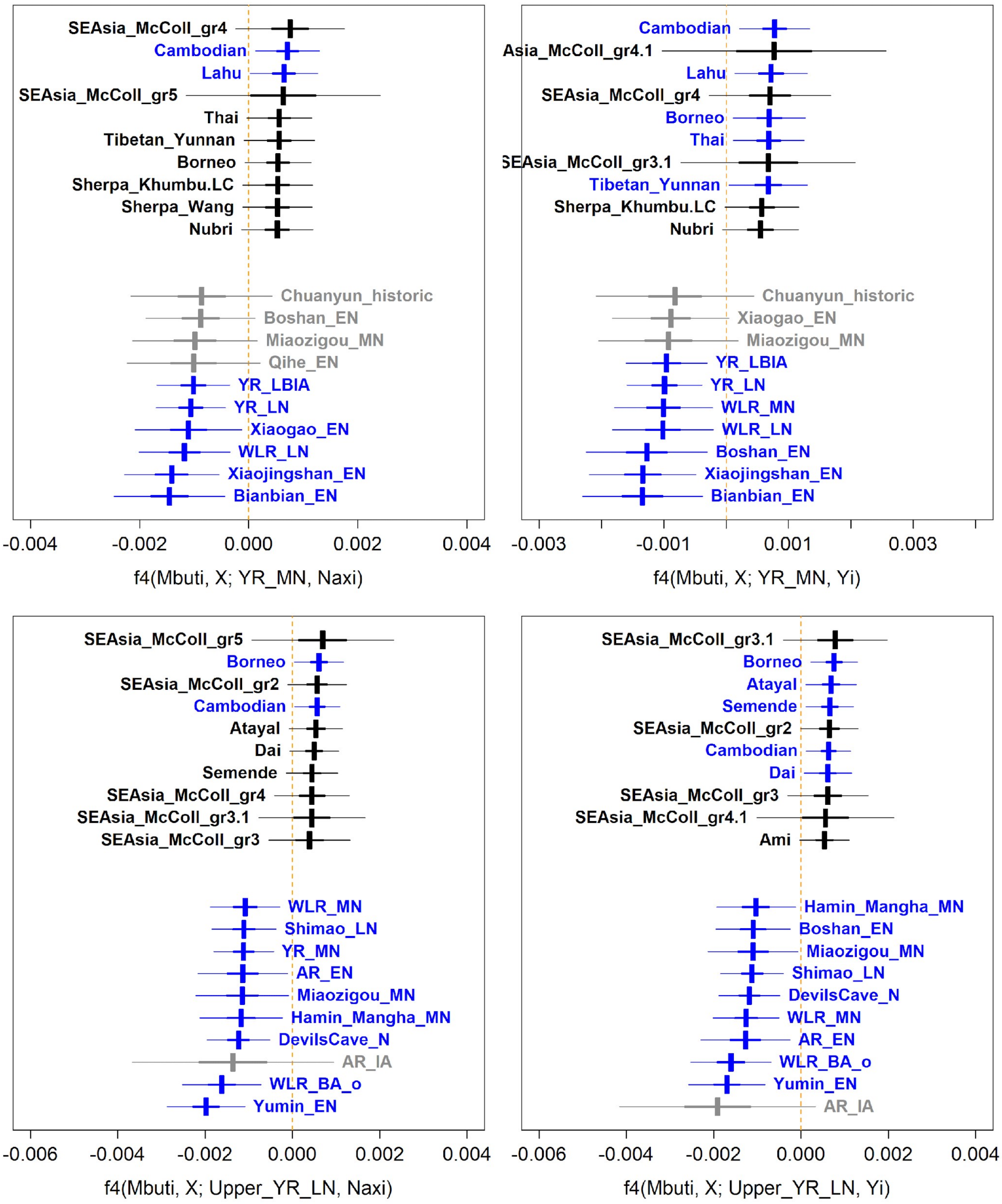

Supplementary Fig. 11. The genetic difference between YR_MN/Upper_YR_LN and Naxi/Yi. We quantify the difference between Upper_YR_LN/YR_MN and Naxi/Yi with f_4(Mbuti, X, YR_MN/Upper_YR_LN, Naxi/Yi), where X's are world-wide ancient and present-day groups. The standard errors are estimated from 5 cM block-jackknifing. Ten most positive and negative signals are presented. Vertical bars mark the point estimate of f_4 statistic. Thick and thin horizontal bars representing ±1 and 3 SEM estimated by 5 cM block jackknifing, respectively.

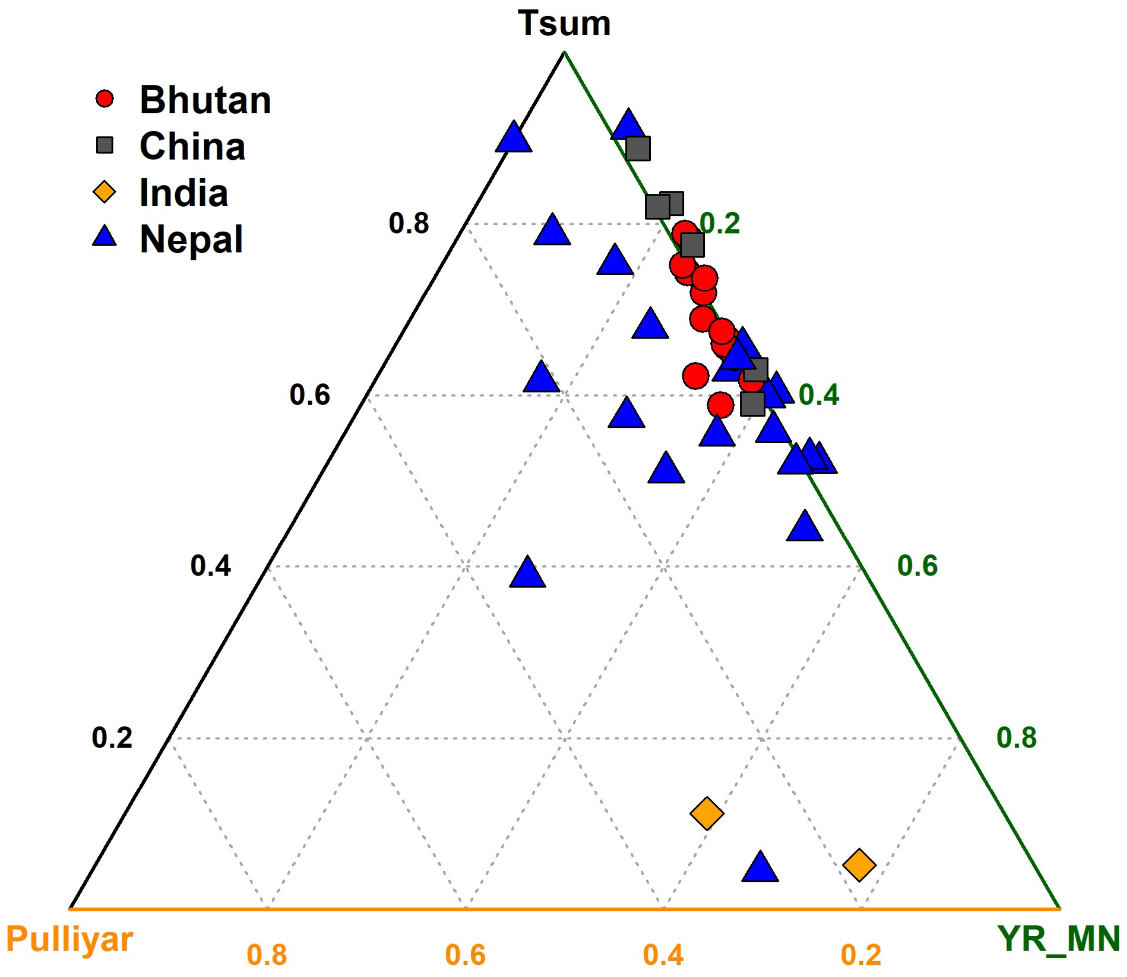

Supplementary Fig. 12. Three-way admixture modeling of the Himalayan populations in the Illumina data set. We used qpAdm to test three-way admixture model, Tsum+YR_MN+Pulliyar. Bhutanese populations have a limited South Asian ancestry (represented by Pulliyar) and overall higher Tibetan ancestry (represented by Tsum) than the Nepalese populations.

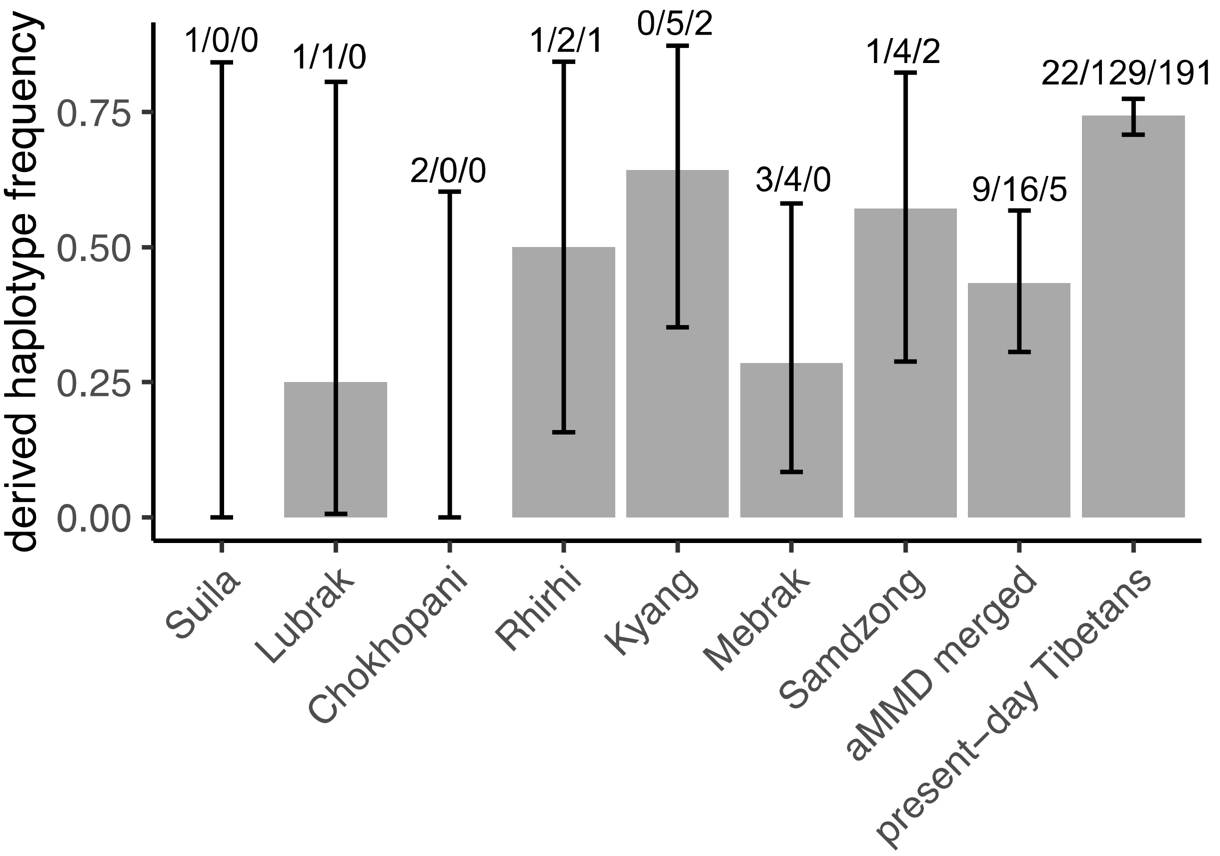

Supplementary Fig. 13. *EPAS1* Allele frequency estimates in aMMD groups. We manually assigned whether an aMMD individual had the derived *EPAS1* haplotype by merging reads covering 19 SNPs tagging the haplotype (Supplementary Tables 8-9). The frequency for each group and its 95% confidence interval was then estimated after removing 1st degree relatives. The height of the bar chart represent the frequency estimate ($\hat{p}$), which was calculated as a/n where a=the number of derived haplotypes and n=the number of all observed haplotypes in each group. The black vertical segments represent 95% Clopper–Pearson confidence interval, computed using the binom.confint function in the binom package v1.1-1 in R. The annotation above each group represents the number of individuals having 0/1/2 derived haplotypes in the respective group.

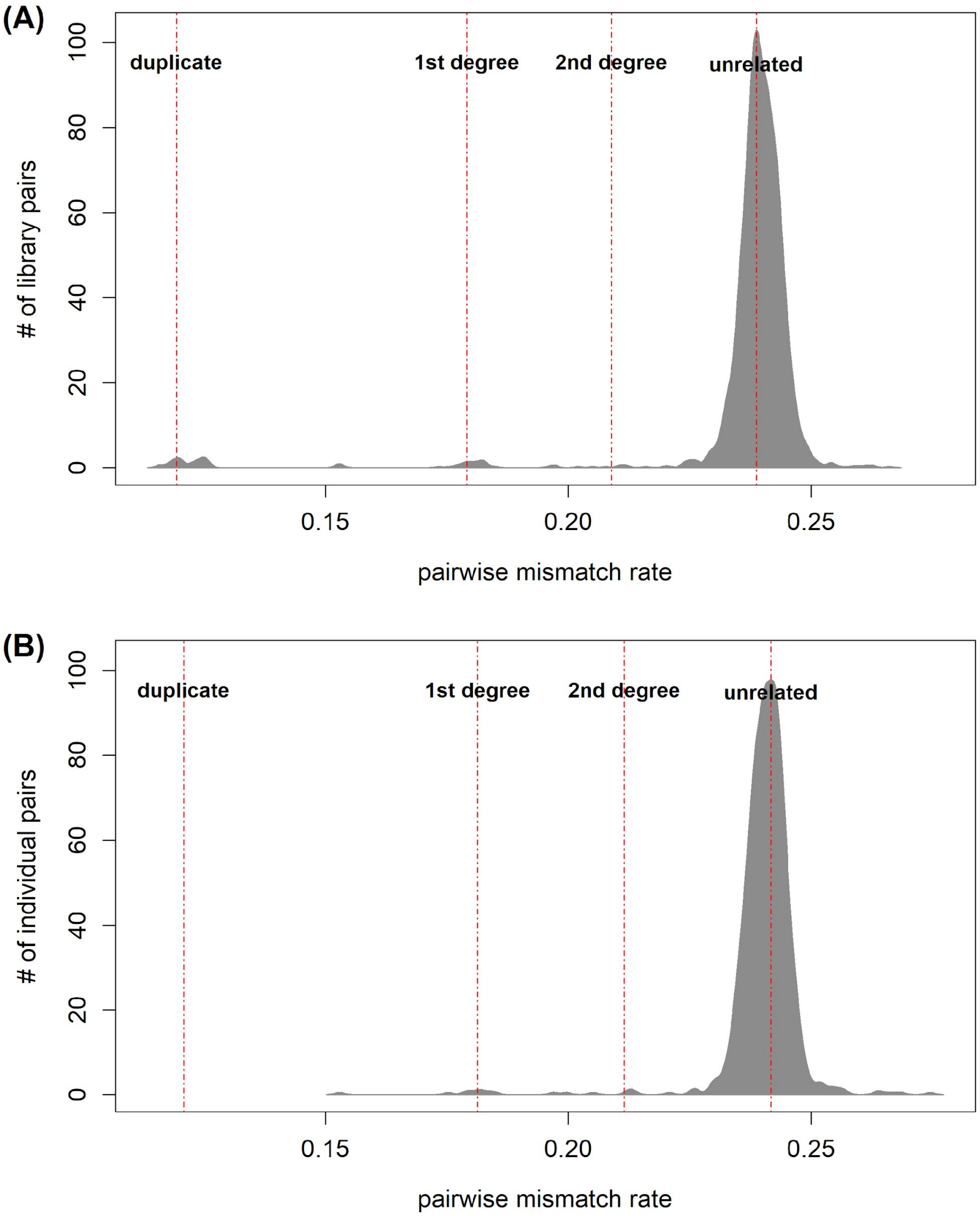

Supplementary Fig. 14. Density plots of pairwise mismatch rates (PMR) between all pairs of aMMD libraries and individuals. (A) We detected libraries from the same individual, either from the same tooth sample or from different samples, as those having PMR values about half of those from the unrelated pairs. (B) After merging libraries from the same individual, we re-calculated PMR between all pairs of individuals, identifying 8 first-degree and 5 second-degree relatives. We calculated density using the "density" function in R v4.0.0. We took the PMR value with the maximum density as the value between the unrelated individuals, and took the value for the duplicates, 1st degree relatives (r=0.5) and 2nd degree relatives (r=0.25) as 1/2, 3/4, and 7/8 of the value for the unrelated pairs, respectively.

Supplementary Table 1. QpWave test for the sister group. We tested whether a pair of aMMD/Nepalese Tibetans for a sister clade against the following outgroups: Mbuti, Onge, Iran_N, Villabruna, Ami, Mixe. To increase the resolution, we also added Lubrak or Chokhopani as an additional outgroup when possible. Small p-values (< 0.05) suggest that the two groups do not form a sister clade. P-values are calculated with a likelihood ratio test comparing a nested model (no significant difference between the two groups) and a nesting one (there is significant difference between the two groups with regard to their affinity to some outgroups).

Refs	Base outgroup	Base+ Lubrak	Base+ Chokhopani	Refs	Base outgroup	Base+ Lubrak	Base+ Chokhopani
Suila;Lubrak	1.26E-01	NA	1.92E-01	Rhirhi;Tsum.LC	1.01E-01	5.90E-16	5.32E-11
Suila;Chokhopani	6.75E-02	1.38E-04	NA	Rhirhi;UpperMustang	3.34E-01	1.19E-17	1.74E-10
Suila;Rhirhi	5.11E-01	6.22E-01	6.32E-01	Rhirhi;UpperMustang.LC	9.74E-03	1.46E-20	3.32E-15
Suila;Kyang	2.36E-01	1.42E-01	1.74E-01	Kyang;Mebrak	7.07E-02	1.51E-02	1.09E-02
Suila;Mebrak	2.41E-01	2.91E-01	3.25E-01	Kyang;Samdzong	7.62E-01	7.09E-01	6.64E-01
Suila;Samdzong	2.35E-01	5.63E-02	2.61E-01	Kyang;Sherpa_Khumbu	9.59E-04	3.60E-23	7.82E-19
Suila;Sherpa_Khumbu	5.03E-02	1.72E-10	1.46E-07	Kyang;Sherpa_Khumbu.LC	1.00E-01	1.85E-16	4.39E-12
Suila;Sherpa_Khumbu.LC	8.67E-02	1.51E-09	1.60E-06	Kyang;Nubri.LC	1.90E-01	2.62E-19	2.60E-09
Suila;Nubri.LC	2.51E-01	4.18E-09	3.19E-05	Kyang;Tsum.LC	2.20E-01	1.05E-16	3.98E-11
Suila;Tsum.LC	3.87E-01	2.00E-08	2.83E-05	Kyang;UpperMustang	7.60E-01	3.02E-20	1.64E-11
Suila;UpperMustang	4.43E-01	1.92E-09	2.82E-05	Kyang;UpperMustang.LC	1.59E-01	2.99E-24	7.27E-18
Suila;UpperMustang.LC	3.52E-02	1.55E-11	1.23E-07	Mebrak;Samdzong	6.25E-01	1.12E-02	4.58E-01
Lubrak;Chokhopani	1.33E-02	NA	NA	Mebrak;Sherpa_Khumbu	5.01E-03	5.45E-33	1.20E-26
Lubrak;Rhirhi	2.58E-01	NA	3.40E-01	Mebrak;Sherpa_Khumbu.LC	2.31E-02	1.32E-26	1.01E-19
Lubrak;Kyang	9.40E-02	NA	2.72E-02	Mebrak;Nubri.LC	8.57E-01	1.17E-27	8.49E-14
Lubrak;Mebrak	5.56E-01	NA	6.55E-01	Mebrak;Tsum.LC	5.72E-01	1.52E-26	1.83E-17
Lubrak;Samdzong	2.64E-01	NA	2.12E-01	Mebrak;UpperMustang	5.14E-02	7.67E-32	3.91E-19
Lubrak;Sherpa_Khumbu	4.63E-02	NA	8.61E-10	Mebrak;UpperMustang.LC	2.68E-02	7.91E-36	1.09E-27
Lubrak;Sherpa_Khumbu.LC	2.41E-01	NA	2.57E-07	Samdzong;Sherpa_Khumbu	1.93E-02	1.82E-18	7.55E-22
Lubrak;Nubri.LC	4.58E-01	NA	2.66E-06	Samdzong;Sherpa_Khumbu.LC	1.53E-01	4.40E-13	1.20E-15
Lubrak;Tsum.LC	3.75E-01	NA	1.02E-07	Samdzong;Nubri.LC	8.48E-01	1.87E-16	2.76E-11
Lubrak;UpperMustang	1.51E-01	NA	5.75E-08	Samdzong;Tsum.LC	7.52E-01	1.41E-13	3.04E-14
Lubrak;UpperMustang.LC	3.48E-01	NA	2.99E-09	Samdzong;UpperMustang	5.77E-01	3.56E-17	9.43E-15
Chokhopani;Rhirhi	2.02E-03	6.10E-08	NA	Samdzong;UpperMustang.LC	2.08E-01	3.96E-22	7.73E-23
Chokhopani;Kyang	6.63E-03	1.85E-05	NA	Sherpa_Khumbu;Sherpa_Khumbu.LC	2.88E-01	2.45E-01	1.42E-01
Chokhopani;Mebrak	4.11E-04	2.12E-10	NA	Sherpa_Khumbu;Nubri.LC	1.09E-01	1.67E-01	3.89E-02
Chokhopani;Samdzong	4.55E-03	1.18E-04	NA	Sherpa_Khumbu;Tsum.LC	5.47E-02	6.16E-02	2.54E-02
Chokhopani;Sherpa_Khumbu	1.73E-02	4.79E-04	NA	Sherpa_Khumbu;UpperMustang	5.08E-03	9.86E-03	5.94E-03
Chokhopani;Sherpa_Khumbu.LC	2.52E-02	2.74E-03	NA	Sherpa_Khumbu;UpperMustang.LC	1.31E-02	1.84E-02	2.51E-02
Chokhopani;Nubri.LC	5.67E-03	2.50E-04	NA	Sherpa_Khumbu.LC;Nubri.LC	3.44E-01	3.33E-01	4.17E-01
Chokhopani;Tsum.LC	1.55E-02	3.27E-03	NA	Sherpa_Khumbu.LC;Tsum.LC	1.94E-01	2.82E-01	2.69E-01
Chokhopani;UpperMustang	4.63E-02	2.23E-03	NA	Sherpa_Khumbu.LC;UpperMustang	9.34E-02	1.30E-01	1.51E-01
Chokhopani;UpperMustang.LC	9.81E-03	6.82E-05	NA	Sherpa_Khumbu.LC;UpperMustang.LC	4.82E-01	2.89E-01	4.21E-01
Rhirhi;Kyang	6.51E-01	3.19E-01	4.16E-01	Nubri.LC;Tsum.LC	9.03E-01	7.57E-01	9.49E-01
Rhirhi;Mebrak	4.12E-01	5.29E-01	5.25E-01	Nubri.LC;UpperMustang	1.44E-01	2.14E-01	1.83E-01
Rhirhi;Samdzong	5.04E-01	6.18E-02	5.52E-01	Nubri.LC;UpperMustang.LC	3.88E-01	4.66E-01	1.23E-01
Rhirhi;Sherpa_Khumbu	1.46E-03	1.96E-19	4.82E-16	Tsum.LC;UpperMustang	2.47E-01	2.79E-01	3.30E-01
Rhirhi;Sherpa_Khumbu.LC	1.45E-02	1.25E-15	9.34E-12	Tsum.LC;UpperMustang.LC	1.80E-01	6.91E-02	9.03E-02
Rhirhi;Nubri.LC	1.94E-01	2.02E-17	2.51E-09	UpperMustang;UpperMustang.LC	1.22E-02	1.63E-02	1.24E-02

Supplementary Table 2. QpAdm-based admixture modeling of aMMD groups. We model Suila/Rhirhi/Kyang/Mebrak/Samdzong as two-way admixture, Lubrak + Ref2 (a set of lowlander groups), using qpAdm. We use Mbuti, Onge, Iran_N, Villabruna, Ami, Mixe, Chokhopani as outgroups. Pdiff refers to the p-value for the significance of the minor ancestry (Ref2), and calculated with a likelihood ratio test comparing a nested model (ref2 has no contribution to the target) and a nesting one (ref2 has some contribution to the target). SEMs are inferred by 5 cM block jackknifing. P-values are calculated with a likelihood ratio test comparing a nested model (the target allele frequency is modeled as a linear combination of the sources) and a nesting one (the target allele frequency can deviate from a linear combination of the sources).

Target	Ref_1	Ref_2	P-value	Pdiff	C1	C2	SEM
Suila	Lubrak	Upper_YR_LN	0.171	0.357	1.107	-0.107	0.126
	Lubrak	YR_MN	0.180	0.320	1.097	-0.097	0.104
	Lubrak	Naga	0.165	0.387	1.104	-0.104	0.130
	Lubrak	Naxi	0.181	0.317	1.100	-0.100	0.107
	Lubrak	Yi	0.193	0.274	1.102	-0.102	0.099
	Lubrak	Pathan	0.274	0.134	0.972	0.028	0.018
	Lubrak	Mala	0.427	0.055	0.954	0.046	0.023
	Lubrak	Pulliyar	0.465	0.046	0.949	0.051	0.025
Rhirhi	Lubrak	Upper_YR_LN	0.330	0.317	1.089	-0.089	0.096
	Lubrak	YR_MN	0.301	0.400	1.061	-0.061	0.077
	Lubrak	Naga	0.293	0.428	1.071	-0.071	0.095
	Lubrak	Naxi	0.305	0.385	1.063	-0.063	0.076
	Lubrak	Yi	0.320	0.341	1.065	-0.065	0.072
	Lubrak	Pathan	0.537	0.102	0.976	0.024	0.014
	Lubrak	Mala	0.676	0.058	0.964	0.036	0.019
	Lubrak	Pulliyar	0.709	0.050	0.961	0.039	0.019
Kyang	Lubrak	Upper_YR_LN	0.015	0.896	0.982	0.018	0.095
	Lubrak	YR_MN	0.015	0.896	0.986	0.014	0.076
	Lubrak	Naga	0.016	0.668	0.959	0.041	0.094
	Lubrak	Naxi	0.015	0.874	0.985	0.015	0.075
	Lubrak	Yi	0.015	1.000	1.000	0.000	0.071
	Lubrak	Pathan	0.062	0.059	0.976	0.024	0.013
	Lubrak	Mala	0.149	0.015	0.960	0.040	0.016
	Lubrak	Pulliyar	0.179	0.011	0.956	0.044	0.017
Mebrak	Lubrak	Upper_YR_LN	0.545	0.745	1.027	-0.027	0.085
	Lubrak	YR_MN	0.546	0.736	1.024	-0.024	0.070
	Lubrak	Naga	0.540	0.781	1.024	-0.024	0.086
	Lubrak	Naxi	0.553	0.686	1.028	-0.028	0.070
	Lubrak	Yi	0.557	0.663	1.029	-0.029	0.067
	Lubrak	Pathan	0.722	0.257	0.986	0.014	0.012
	Lubrak	Mala	0.737	0.239	0.981	0.019	0.016
	Lubrak	Pulliyar	0.724	0.254	0.981	0.019	0.017
Samdzong	Lubrak	Upper_YR_LN	0.139	1.000	1.006	-0.006	0.092
	Lubrak	YR_MN	0.139	1.000	1.005	-0.005	0.073
	Lubrak	Naga	0.139	0.975	0.991	0.009	0.091
	Lubrak	Naxi	0.139	1.000	1.006	-0.006	0.072
	Lubrak	Yi	0.141	0.858	1.013	-0.013	0.068
	Lubrak	Pathan	0.287	0.145	0.982	0.018	0.012
	Lubrak	Mala	0.414	0.069	0.971	0.029	0.016
	Lubrak	Pulliyar	0.436	0.062	0.969	0.031	0.016

Supplementary Table 3. QpAdm-based admixture modeling of Chokhopani. We model Chokhopani as two-way admixture, Lubrak/Suila + Ref2 (a set of lowlander groups), using qpAdm. We use Mbuti, Onge, Iran_N, Villabruna, Ami, Mixe, Lubrak/Suila as outgroups: i.e., we add Lubrak into the outgroup when we use Suila as a reference, and vice versa. Pdiff refers to the p-value for the significance of the minor ancestry (Ref2), and calculated with a likelihood ratio test comparing a nested model (ref2 has no contribution to the target) and a nesting one (ref2 has some contribution to the target). SEMs are inferred by 5 cM block jackknifing. P-values are calculated with a likelihood ratio test comparing a nested model (the target allele frequency is modeled as a linear combination of the sources) and a nesting one (the target allele frequency can deviate from a linear combination of the sources).

Target	Ref_1	Ref_2	P-value	Pdiff	C1	C2	SEM
Chokhopani	Lubrak	Upper_YR_LN	8.38E-05	0.021	0.753	0.247	0.112
	Lubrak	YR_MN	1.11E-04	0.015	0.780	0.220	0.088
	Lubrak	Naga	3.67E-04	0.003	0.693	0.307	0.100
	Lubrak	Naxi	2.25E-04	0.006	0.766	0.234	0.080
	Lubrak	Yi	1.39E-04	0.011	0.799	0.201	0.075
	Lubrak	Pathan	9.99E-06	0.442	0.989	0.011	0.014
	Lubrak	Mala	2.86E-05	0.087	0.968	0.032	0.018
	Lubrak	Pulliyar	4.63E-05	0.044	0.961	0.039	0.019
	Suila	Upper_YR_LN	**0.095**	2.84E-05	0.643	0.357	0.069
	Suila	YR_MN	**0.083**	3.43E-05	0.670	0.330	0.064
	Suila	Naga	**0.198**	9.53E-06	0.598	0.402	0.070
	Suila	Naxi	**0.150**	1.45E-05	0.660	0.340	0.061
	Suila	Yi	**0.124**	1.90E-05	0.686	0.314	0.059
	Suila	Pathan	1.11E-04	0.234	1.020	-0.020	0.017
	Suila	Mala	7.05E-05	0.529	1.015	-0.015	0.023
	Suila	Pulliyar	6.08E-05	0.738	1.009	-0.009	0.024

Supplementary Table 4. Summary statistics for the key features of the qpGraph analysis. (A) aMMD groups have extra affinity to Devil's Gate compared to YR_MN. We report z-scores of f4(Ami, DevilsGate, YR_MN, aMMD) for the same topologies in Supplementary Fig. 7. For each aMMD group the z-score is > 2, suggesting that YR_MN fails to mimic the primary source to due to extra affinity to Ancient Northeast Asian in aMMD groups. (B) Deep lineage ancestry proportions and z-scores for the two best admixture graph topologies. We report the worst z-score and the ancestry proportion derived from the deep ancestry in the two best topologies (Supplementary Fig. 7) for each aMMD group.

(A) f4(Ami, DevilsGate, YR_MN, aMMD)

aMMD	topology #1 z-scores	topology #2 z-scores
Lubrak	2.548	2.546
Chokhopani	2.413	2.353
Rhirhi	3.935	3.891
Samdzong	2.414	2.390
Mebrak	4.353	4.318
Kyang	3.431	3.401
Suila	2.961	2.942

(B) qpGraph z-scores for the 2 best topologies

aMMD	Topology	Deep lineage proportion	Worst z-score
Lubrak	1	8.3%	2.878
Chokhopani	1	17.8%	2.326
Rhirhi	1	19.9%	2.248
Samdzong	1	16.2%	2.316
Mebrak	1	10.6%	-2.490
Kyang	1	15.0%	2.316
Suila	1	17.7%	2.300
Lubrak	2	8.3%	2.878
Chokhopani	2	11.6%	2.326
Rhirhi	2	15.4%	2.248
Samdzong	2	14.2%	2.316
Mebrak	2	10.1%	2.316
Kyang	2	12.6%	2.316
Suila	2	14.5%	2.300

Supplementary Table 5. QpAdm modeling of the Tibetan cline. We model Tibeto-Burman speaking groups as Tsum + Upper_YR_LN/YR_MN using qpAdm with Lubrak as an extra outgroup to increase the resolution on differentiating the Tibetan lineage and lowland ancestries. P-values are calculated with a likelihood ratio test comparing a nested model (the target allele frequency is modeled as a linear combination of the sources) and a nesting one (the target allele frequency can deviate from a linear combination of the sources). Pdiff refers to the p-value for the significance of the minor ancestry (either ref1 or ref2), and calculated with a likelihood ratio test comparing a nested model (the minor source has no contribution to the target) and a nesting one (it has some contribution to the target). SEMs are inferred by 5 cM block jackknifing.

Target	Ref2	P-value	Pdiff	C1	C2	SEM
Sherpa_Khumbu	Upper_YR_LN	1.36E-01	5.94E-02	0.875	0.125	0.066
Sherpa_Khumbu.LC	Upper_YR_LN	3.21E-01	2.17E-01	0.916	0.084	0.067
UpperMustang	Upper_YR_LN	2.21E-01	5.20E-01	1.042	-0.042	0.068
UpperMustang.LC	Upper_YR_LN	3.06E-01	1.81E-02	0.859	0.141	0.057
Nubri.LC	Upper_YR_LN	7.56E-01	3.89E-01	0.945	0.055	0.062
Tibetan_Chamdo	Upper_YR_LN	2.15E-01	2.49E-13	0.507	0.493	0.053
Tibetan_Gangcha	Upper_YR_LN	4.64E-18	7.48E-06	0.380	0.620	0.102
Tibetan_Gannan	Upper_YR_LN	1.16E-14	5.03E-02	-0.187	1.187	0.132
Tibetan_Lhasa	Upper_YR_LN	5.15E-01	2.17E-05	0.761	0.239	0.053
Tibetan_Nagqu	Upper_YR_LN	9.68E-01	5.57E-12	0.582	0.418	0.055
Tibetan_Shannan	Upper_YR_LN	5.59E-01	1.04E-02	0.853	0.147	0.055
Tibetan_Shigatse	Upper_YR_LN	1.14E-01	3.95E-05	0.766	0.234	0.056
Tibetan_Xinlong	Upper_YR_LN	5.96E-02	4.15E-05	-0.287	1.287	0.087
Tibetan_Xunhua	Upper_YR_LN	4.69E-09	5.46E-03	0.246	0.754	0.094
Tibetan_Yajiang	Upper_YR_LN	4.00E-01	7.53E-05	0.277	0.723	0.059
Tibetan_Yunnan	Upper_YR_LN	1.65E-01	9.71E-03	0.218	0.782	0.079
Qiang_Danba	Upper_YR_LN	4.42E-01	2.05E-02	-0.162	1.162	0.078
Qiang_Daofu	Upper_YR_LN	7.41E-01	2.47E-01	0.079	0.921	0.065
Tamang	Upper_YR_LN	4.38E-22	0.00E+00	2.160	-1.160	0.254
Gurung	Upper_YR_LN	6.28E-12	1.00E+00	1.025	-0.025	0.129
Naxi	Upper_YR_LN	1.99E-02	4.24E-02	-0.151	1.151	0.084
Yi	Upper_YR_LN	8.99E-03	2.09E-05	-0.300	1.300	0.090
Naga	Upper_YR_LN	3.90E-01	6.18E-02	0.132	0.868	0.066
Sherpa_Khumbu	YR_MN	6.34E-02	2.23E-01	0.930	0.070	0.058
Sherpa_Khumbu.LC	YR_MN	2.28E-01	4.93E-01	0.959	0.041	0.058
UpperMustang	YR_MN	2.47E-01	3.90E-01	1.047	-0.047	0.057
UpperMustang.LC	YR_MN	2.99E-01	1.90E-02	0.879	0.121	0.049
Nubri.LC	YR_MN	7.35E-01	4.37E-01	0.957	0.043	0.054
Tibetan_Chamdo	YR_MN	4.23E-03	1.44E-15	0.593	0.407	0.050
Tibetan_Gangcha	YR_MN	3.96E-19	3.10E-12	0.552	0.448	0.071
Tibetan_Gannan	YR_MN	1.68E-11	3.18E-01	0.082	0.918	0.086
Tibetan_Lhasa	YR_MN	2.20E-01	9.46E-05	0.807	0.193	0.048
Tibetan_Nagqu	YR_MN	1.87E-01	1.61E-10	0.665	0.335	0.049
Tibetan_Shannan	YR_MN	3.15E-01	3.24E-02	0.894	0.106	0.048
Tibetan_Shigatse	YR_MN	9.68E-03	1.14E-03	0.842	0.158	0.049
Tibetan_Xinlong	YR_MN	6.92E-01	1.21E-01	-0.104	1.104	0.073
Tibetan_Xunhua	YR_MN	4.11E-08	1.88E-05	0.336	0.664	0.077
Tibetan_Yajiang	YR_MN	4.84E-01	1.32E-08	0.379	0.621	0.050
Tibetan_Yunnan	YR_MN	5.49E-01	4.25E-05	0.313	0.687	0.065
Qiang_Danba	YR_MN	4.30E-01	9.03E-01	-0.010	1.010	0.070
Qiang_Daofu	YR_MN	1.26E-01	2.78E-03	0.196	0.804	0.057
Tamang	YR_MN	8.15E-24	0.00E+00	2.138	-1.138	0.256
Gurung	YR_MN	6.18E-12	1.00E+00	0.984	0.016	0.110
Naxi	YR_MN	3.75E-01	7.52E-01	0.023	0.977	0.069
Yi	YR_MN	4.83E-01	8.92E-02	-0.114	1.114	0.074
Naga	YR_MN	2.30E-01	5.32E-04	0.247	0.753	0.061

Supplementary Table 6. QpAdm modeling of Naga. We model Naga as a two-way admixture of Tsum + another East Asian/South Asian source. We use Lubrak as an extra outgroup on top of the six base outgroups. P-values are calculated with a likelihood ratio test comparing a nested model (the target allele frequency is modeled as a linear combination of the sources) and a nesting one (the target allele frequency can deviate from a linear combination of the sources). Pdiff refers to the p-value for the significance of the minor ancestry (either ref1 or ref2), and calculated with a likelihood ratio test comparing a nested model (the minor source has no contribution to the target) and a nesting one (it has some contribution to the target). SEMs are inferred by 5 cM block jackknifing.

Target	Ref$_2$	P-value	Pdiff	C1	C2	SEM
Naga	Upper_YR_LN	3.90E-01	6.18E-02	0.132	0.868	0.066
	**YR_MN**	**2.30E-01**	**5.32E-04**	**0.247**	**0.753**	**0.061**
	Naxi	4.92E-01	6.52E-06	0.224	0.776	0.045
	Yi	1.70E-01	7.05E-14	0.316	0.684	0.039
	Pathan	8.71E-29	9.10E-15	1.066	-0.066	0.009
	Mala	6.88E-29	1.04E-14	1.088	-0.088	0.012

Supplementary Table 7. QpAdm modeling of Tamang and Gurung. We used qpAdm to test three-way admixture model, Tsum + Ref2(lowland East Asian) + Ref3(South Asian), on Tamang and Gurung. We use Lubrak as an extra outgroup on top of the six base outgroups. P-values are calculated with a likelihood ratio test comparing a nested model (the target allele frequency is modeled as a linear combination of the sources) and a nesting one (it can deviate from a linear combination of the sources). SEMs are inferred by 5 cM block jackknifing.

Target	Ref_2	Ref_3	P-value	C1	C2	C3	SEM1	SEM2	SEM3
Tamang	Upper_YR_LN	Pathan	2.08E-08	0.655	0.216	0.129	0.102	0.095	0.010
	Upper_YR_LN	Mala	6.74E-01	0.547	0.263	0.190	0.061	0.057	0.011
	Upper_YR_LN	Pulliyar	9.96E-01	0.557	0.244	0.198	0.060	0.055	0.011
	YR_MN	Pathan	4.22E-08	0.687	0.186	0.127	0.077	0.072	0.009
	YR_MN	Mala	4.73E-01	0.598	0.215	0.186	0.053	0.049	0.011
	YR_MN	Pulliyar	8.13E-01	0.606	0.199	0.195	0.053	0.048	0.011
	Naga	Pathan	5.09E-08	0.585	0.282	0.133	0.113	0.106	0.010
	Naga	Mala	8.71E-01	0.490	0.317	0.193	0.067	0.063	0.010
	Naga	Pulliyar	8.50E-01	0.525	0.276	0.199	0.067	0.063	0.011
	Naxi	Pathan	9.14E-08	0.657	0.212	0.131	0.069	0.064	0.009
	Naxi	Mala	6.99E-01	0.573	0.237	0.190	0.053	0.049	0.010
	Naxi	Pulliyar	7.27E-01	0.595	0.207	0.197	0.054	0.049	0.011
	Yi	Pathan	4.92E-08	0.701	0.171	0.128	0.062	0.057	0.009
	Yi	Mala	5.53E-01	0.604	0.206	0.190	0.050	0.045	0.011
	Yi	Pulliyar	7.05E-01	0.619	0.184	0.198	0.049	0.044	0.011
Gurung	Upper_YR_LN	Pathan	3.70E-03	0.613	0.322	0.065	0.084	0.080	0.009
	Upper_YR_LN	Mala	5.54E-01	0.549	0.356	0.096	0.073	0.068	0.011
	Upper_YR_LN	Pulliyar	7.65E-01	0.550	0.350	0.101	0.072	0.067	0.012
	YR_MN	Pathan	2.50E-03	0.683	0.255	0.062	0.068	0.065	0.008
	YR_MN	Mala	2.40E-01	0.630	0.279	0.091	0.063	0.058	0.011
	YR_MN	Pulliyar	3.23E-01	0.631	0.273	0.095	0.063	0.058	0.011
	Naga	Pathan	1.49E-02	0.509	0.420	0.071	0.097	0.092	0.009
	Naga	Mala	6.68E-01	0.474	0.427	0.100	0.084	0.079	0.011
	Naga	Pulliyar	6.04E-01	0.491	0.406	0.103	0.084	0.079	0.011
	Naxi	Pathan	8.19E-03	0.642	0.291	0.067	0.065	0.061	0.008
	Naxi	Mala	3.82E-01	0.601	0.304	0.096	0.062	0.057	0.011
	Naxi	Pulliyar	3.62E-01	0.611	0.290	0.099	0.062	0.057	0.011
	Yi	Pathan	6.51E-03	0.682	0.252	0.066	0.059	0.055	0.009
	Yi	Mala	3.69E-01	0.638	0.266	0.096	0.057	0.051	0.011
	Yi	Pulliyar	3.94E-01	0.644	0.256	0.099	0.057	0.051	0.012

Supplementary Table 8. *EGLN1* and *EPAS1* allele counts and frequency estimates. We manually assigned whether an aMMD individual had the derived *EPAS1* haplotype by merging reads covering 19 SNPs tagging the haplotype (Supplementary Table 9). The derived haplotype frequency for each group was then estimated after removing 1st degree relatives. For the two *EGLN1* derived SNPs, we follow Mathieson et al 2015 to obtain maximum-likelihood allele frequency estimates for each aMMD group.

	EGLN1 rs12097901		*EGLN1* rs186996510		*EPAS1*		
Individual	ancestral / derived base count	derived allele frequency	ancestral / derived base count	derived allele frequency	ancestral / derived base count	genotype	derived allele frequency
U1	0/0	Suila	0/0	Suila	43/0	0	Suila
		N/A		N/A			0.000
L1	0/0	Lubrak	0/0	Lubrak	10/0	0	Lubrak
L2	0/0	N/A	0/0	N/A	13/11	1	0.250
C1	4/2	Chokhopani	0/2	Chokhopani	116/0	0	Chokhopani
CNE1	2/0	0.293	0/0	1.000	111/0	0	0.000
KM4	0/6		0/4		138/106	1	
KS20_KS25	0/0		1/1		132/136	1	
KS21_KS23_KS4	0/2		0/2		125/134	1	
KS26	0/1		0/5		2/158	2	
KS5	2/3		2/0		87/101	1	
KS8	0/1	Kyang	0/2	Kyang	1/61	2	Kyang
KS9	0/0	0.865	0/0	0.779	13/12	1	0.643
M2113	0/1		0/2		196/1	0	
M241	0/0		0/0		1/0		
M295	0/0		0/1		70/68	1	
M354	0/0		0/1		40/0	0	
M368	0/9		0/9		109/123	1	
M4580	0/0		0/0		12/9	1	
M4681	0/0	Mebrak	0/0	Mebrak	49/56	1	Mebrak
M63_M339_M359	0/1	1.000	0/0	1.000	77/0	0	0.286
R1	0/0		0/0		64/0	0	
R2_R7	2/0		0/0		72/79	1	
R5	0/0	Rhirhi	0/0	Rhirhi	9/6	1	Rhirhi
R8	0/0	0.000	0/0	N/A	0/38	2	0.500
S10_S13	0/1		0/3		155/1	0	
S143_S173	0/0		0/0		1/215	2	
S153_S183	0/0		0/0		45/42	1	
S18_S20_S21_S22	0/0		0/1		81/79	1	
S29_S30	0/0		0/0		0/0		
S35	0/4		0/10		38/42	1	
S36	0/0		0/0		0/0		
S41	0/0	Samdzong	0/5	Samdzong	2/40	2	Samdzong
S8	0/0	1.000	1/0	0.870	91/51	1	0.571
		All		All			All
		0.765		0.873			0.433

Supplementary Table 9. List of 19 *EPAS1* tagging SNPs. We present their rs number, chromosomal position in GRCh37, and the read counts for the reference and alternative alleles across all aMMD individuals.

SNP	Chromosome	Position	Reference allele	Alternative allele	Ref allele count	Alt allele count
rs115321619	2	46,567,916	G	A	31	29
rs73926263	2	46,568,680	A	G	57	38
rs73926264	2	46,569,017	A	G	78	58
rs73926265	2	46,569,770	G	A	87	77
rs55981512	2	46,570,342	G	A	178	147
rs149306391	2	46,571,017	C	G	95	78
rs374487821	2	46,571,435	G	C	90	84
rs188801636	2	46,577,251	T	C	30	12
rs375554942	2	46,579,689	A	G	250	215
rs189807021	2	46,583,581	G	A	148	167
rs372272284	2	46,584,859	A	G	84	45
rs150877473	2	46,588,019	C	G	173	92
rs142826801	2	46,588,331	G	C	103	101
rs74898705	2	46,589,032	C	T	149	113
rs141366568	2	46,594,122	A	G	58	44
rs116062164	2	46,597,756	A	C	69	54
rs141426873	2	46,598,025	C	G	87	111
rs116611511	2	46,600,030	A	G	59	59
rs369097672	2	46,600,358	A	G	77	46

Supplementary Table 10. Top 10 signals from the genome-wide selection scan. In each East Asian LD block, we obtain a window with the highest z-score, and we report windows with the top 10 signals.

Chromosome	median position	# of SNP	z-score	gene	
2	46510595	1135	14.095	*EPAS1*	
1	231370923	857	12.424	*EGLN1*	
6	72962760	648	8.856	*RIMS1*	
7	3385485	900	8.272		regulating synaptic membrane exocytosis 1
5	128832041	491	8.169		
2	26030595	476	7.403	*KIF3C*	kinesin family member 3C
4	64576862	465	7.094		
5	42402041	279	6.942		
2	213860595	492	6.727		

References

1. Banskota, K. & Sharma, B. Tourism for mountain community development: Case study report on the Annapurna and Gorkha regions of Nepal. International Centre for Integrated Mountain Development. (1995).
2. Reimer, P. J. *et al.* The IntCal20 Northern Hemisphere radiocarbon age calibration curve (0-55 CAL kBP). *Radiocarbon* **62**, 11-33 (2020).
3. Tiwari, D. Cave burials from western Nepal, Mustang. *Anc. Nepal* **136**, 51–75 (1984).
4. Simons, A., Schön, W. & Shrestha, S. Preliminary report on the 1992 campaign of the Institute of Prehistory of the University of Cologne. *Anc. Nepal* **136**, 51–75 (1994).
5.. Aldenderfer, M. & Eng, J. T. Death and burial among two ancient hgh-altitude communities of Nepal. in *A Companion to South Asia in the Past* 374–397 (John Wiley & Sons, Inc, 2016).
6. Angela Simons (Ed.): *Hirten im Himalaya – Prähistorische Mumien im Höhlengrab Mebrak 63 (Mustang/Nepal)*. Heidelberg: Propylaeum, 2020 (Archäologische Berichte, Vol. 31).
7. Eng, J. & Aldenderfer, M. Interdisciplinary Approaches to reconstructing early population history in the high Himalayas of Nepal. *Bioarch Int.* **4,** 1-20 (2020)
8. Aldenderfer, M. Variation in mortuary practice on the early Tibetan plateau and the high Himalayas. *J. Int. Soc. Bon Stud.* **1**, 273–298 (2013).
9. Jeong, C. *et al.* Long-term genetic stability and a high-altitude East Asian origin for the peoples of the high valleys of the Himalayan arc. *Proc. Natl. Acad. Sci. U. S. A.* **113**, 7485–7490 (2016).
