# Supplementary material for: Ancient genomes from the Himalayas illuminate the genetic history of Tibetans and their Tibeto-Burman speaking neighbors
Source: Nat Commun. 2022 Mar 8;13:1203. doi: 10.1038/s41467-022-28827-2 (PMC8904508; doi:10.1038/s41467-022-28827-2)
Supplement: Supplementary file 3 — Description of Additional Supplementary Files [file 41467_2022_28827_MOESM3_ESM.pdf]

Description of Additional Supplementary Files

File Name: Supplementary Data 1
Description: Per library sequencing summary information of aMMD genomes generated in this study. We report sequencing strategy (WGS/1240K capture/50K capture), UDG treatment, damage patterns, and contamination estimates for each library.

File Name: Supplementary Data 2
Description: Per individual information of aMMD genomes generated in this study. We report per individual information including genetic sex, coverage, and uniparental haplogroup, after merging library duplicates (Supplementary Data 4). Six individuals marked in yellow shades are previously published genome data. Four individuals highlighted in red color failed to pass our quality control and were not used in our analysis further.

File Name: Supplementary Data 3
Description: Accelerator Mass Spectrometry (AMS) dates of the aMMD sites investigated in this study. We report 15 new AMS dates as well as previously published dates for each of the seven aMMD sites.

File Name: Supplementary Data 4
Description: Library duplicates and relatedness analysis. (A) We report PMR between duplicate pairs of libraries (B) We re-calculate PMR per individual, and identify 8 first-degree and 5 second-degree relatives. We report these PMR and IBD coefficients from the lcMLkin v0.5.0 program, which distinguished the first-degree relatives into five parent-offspring pairs and three full siblings.

File Name: Supplementary Data 5
Description: Populations and individuals in the HumanOrigins dataset.

File Name: Supplementary Data 6
Description: Present-day populations in the Illumina dataset.

File Name: Supplementary Data 7
Description: Genetic differences between Lubrak and Chokhopani. We calculate f_4(Mbuti, East Asian; Chokhopani, Lubrak) to test whether an ancient or a present-day East Asian group are genetically closer to one than the other. Standard error measures (SEM) are calculated by 5 cM block jackknifing as implemented in the qpDstat program. Z scores are calculated by dividing f_4 statistic by SEM.

File Name: Supplementary Data 8
Description: Genetic differences between aMMD groups and present-day Nepalese Tibetans. We tested if any ancient or present-day lowland East Asian groups are closer to aMMD than to present-day Sherpa/Tibetans using f_4(Mbuti, East Asian; Chokhopani/Lubrak, present-day Nepalese Sherpa/Tibetan). SEMs are calculated by 5 cM block jackknifing as implemented in the qpDstat program. Z scores are calculated by dividing f_4 statistic by SEM.

File Name: Supplementary Data 9
Description: QpAdm modeling of Himalayan populations from the Illumina dataset. We used qpAdm to test three-way admixture model, Tsum + Naga/YR_MN + Ref3(South Asian), on populations in Bhutan, Nepal, and Tibet reported in Arciero et al 2018. We use Lubrak as an extra outgroup on top of the six base outgroups. P-values are calculated with a likelihood ratio test comparing a nested model (the target allele frequency is modeled as a linear combination of the sources) and a nesting one (it can deviate from a linear combination of the sources). SEMs are inferred by 5 cM block jackknifing.

File Name: Supplementary Data 10
Description: Top 100 variants in the peak window overlapping the *EGLN1* region from the genome-wide selection scan. We report allele frequencies of the 100 variants in present-day Tibetans, 1000 Genomes project Han Chinese from Beijing (CHB), and aMMD, along with LD between these variants and the two nonsynonymous SNPs rs12097901 and rs186996510. REF/ALT/ANC represent reference, alternative, and ancestral allele, respectively. ALT/Total represents the number of alternative and total alleles observed in aMMD individuals.

File Name: Supplementary Data 11
Description: Windows in selection scan with z-score > 4 and overlapping PBS top SNPs. We overlap PBS lead SNPs with windows with outgroup-f_3 z-scores > 4, and report these windows along with PBS top SNPs and genes.
