# Supplementary material for: Ancient genomes from the Himalayas illuminate the genetic history of Tibetans and their Tibeto-Burman speaking neighbors
Source: Nat Commun. 2022 Mar 8;13:1203. doi: 10.1038/s41467-022-28827-2 (PMC8904508; doi:10.1038/s41467-022-28827-2)
Supplement: Supplementary file 5 — Reporting Summary [file 41467_2022_28827_MOESM5_ESM.pdf]

Corresponding author(s): Choongwon Jeong

Last updated by author(s): Feb 13, 2022

Reporting Summary

Nature Research wishes to improve the reproducibility of the work that we publish. This form provides structure for consistency and transparency in reporting. For further information on Nature Research policies, see our Editorial Policies and the Editorial Policy Checklist.

Statistics

For all statistical analyses, confirm that the following items are present in the figure legend, table legend, main text, or Methods section.

n/a	Confirmed	
☐	☒	The exact sample size (*n*) for each experimental group/condition, given as a discrete number and unit of measurement
☐	☒	A statement on whether measurements were taken from distinct samples or whether the same sample was measured repeatedly
☐	☒	The statistical test(s) used AND whether they are one- or two-sided<br>*Only common tests should be described solely by name; describe more complex techniques in the Methods section.*
☒	☐	A description of all covariates tested
☐	☒	A description of any assumptions or corrections, such as tests of normality and adjustment for multiple comparisons
☐	☒	A full description of the statistical parameters including central tendency (e.g. means) or other basic estimates (e.g. regression coefficient) AND variation (e.g. standard deviation) or associated estimates of uncertainty (e.g. confidence intervals)
☐	☒	For null hypothesis testing, the test statistic (e.g. *F*, *t*, *r*) with confidence intervals, effect sizes, degrees of freedom and *P* value noted<br>*Give P values as exact values whenever suitable.*
☒	☐	For Bayesian analysis, information on the choice of priors and Markov chain Monte Carlo settings
☒	☐	For hierarchical and complex designs, identification of the appropriate level for tests and full reporting of outcomes
☒	☐	Estimates of effect sizes (e.g. Cohen's *d*, Pearson's *r*), indicating how they were calculated

Our web collection on statistics for biologists contains articles on many of the points above.

Software and code

Policy information about availability of computer code

Data collection	Illumina sequence data were processed using the following programs to obtain genotype data used in the analysis: AdapterRemoval v2.2.0, BWA v0.7.12, samtools v1.9, DeDup v0.12.2, bamUtils v1.0.14, pileupCaller in the sequenceTools v1.4.0.5(https://github.com/stschiff/sequenceTools), mapDamage v2.0.9, ANGSD v0.910, Schmutzi v1.5.1. These programs are publicly available.
Data analysis	Population genetic data analysis in this study was performed using the following publicly available programs: DATES v753, HaploGrep2, smartpca v16000, PLINK v1.90, lcMLkin v0.5.0, ADMIXTURE v1.3.0, qp3Pop v650, qpDstat v970, qpWave v1200, qpAdm v1201, qpGraph v7365. Non-default parameters used in our analysis are described in the Methods section. The base map in Figures 1 and 5 was created in R v4.0.0 using publicly available libraries mapdata v2.3.0 and elevatr v0.3.4. The base map in Supplementary Figures 3 and 4 was created in R v4.0.5 using publicly available map information from the sf v0.9-8 and rnathralearth v0.2.0 packages. Calibration of AMS 14C dating results was done by Calib 8.2 using the IntCal20 database.

For manuscripts utilizing custom algorithms or software that are central to the research but not yet described in published literature, software must be made available to editors and reviewers. We strongly encourage code deposition in a community repository (e.g. GitHub). See the Nature Research guidelines for submitting code & software for further information.

Data

Policy information about availability of data

All manuscripts must include a data availability statement. This statement should provide the following information, where applicable:

- Accession codes, unique identifiers, or web links for publicly available datasets
- A list of figures that have associated raw data
- A description of any restrictions on data availability

The raw DNA sequences (FASTQ) and the alignment data (BAM) reported in this paper have been deposited in the European Nucleotide Archive (ENA) under the

accession number PRJEB41752 [https://www.ebi.ac.uk/ena/browser/view/PRJEB41752?show=reads]. The genotype data for the 1240K and HumanOrigins panels have been deposited in the Edmond Data Repository of the Max Planck Society [https://edmond.mpdl.mpg.de/imeji/collection/H8mcNv5pbSen6sLg?q=]. The 15 new AMS dates reported in this study, their associated lab codes, and their corresponding lab protocols are provided in Supplementary Data 3. Previously published genome-wide data of ancient individuals used in this study are listed in Supplementary Data 5 and are available via the following sources: 1) the combined genotype data for the 1240K panel SNPs provided by the Reich Lab [https://reich.hms.harvard.edu/datasets], 2) BAM files of the Devil's Gate Cave individuals in the ENA under the accession number PRJEB29700 [https://www.ebi.ac.uk/ena/browser/view/PRJEB29700?show=reads], and 3) the genotype data for the 1240K panel SNPs of ancient individuals from China in the Genome Sequence Archive in the Beijing Institute of Genomics Data Center under the accession number HRA000123 [https://ngdc.cncb.ac.cn/gsa-human/browse/HRA000123].

Field-specific reporting

Please select the one below that is the best fit for your research. If you are not sure, read the appropriate sections before making your selection.

☐ Life sciences ☐ Behavioural & social sciences ☒ Ecological, evolutionary & environmental sciences

For a reference copy of the document with all sections, see nature.com/documents/nr-reporting-summary-flat.pdf

Ecological, evolutionary & environmental sciences study design

All studies must disclose on these points even when the disclosure is negative.

Study description	This study includes production of whole genome or genome-wide sequencing of 33 ancient individuals from the Mustang and Manang District (MMD) region in Nepal, out of 54 skeletal elements screened, ranging between 1494 BC and 952 CE. Sequencing coverage ranges up to 0.5-66x for whole genomes and 0.5-13.2x for the 1240K panel. Including samples from a previous study, a total of 38 ancient genomes come from seven different high altitude sites from different time periods: Suila (n=1; 1494-1317 BCE), Lubrak (n=2; 1269-1123 BCE), Chokhopani (n=3; 801-770 BCE), Rhirhi (n=4; 805-767 BCE), Kyang (n=7; 695-206 BCE), Mebrak (n=9; 500 BCE-1 CE), and Samdzong (n=12; 450-650 CE).
Research sample	Research samples are composed of 38 ancient genomes (for 33 of which we produced new data) from seven archaeological sites the MMD region, Nepal. These seven sites represent different time periods in the region, constituting a time series: Suila (n=1; 1494-1317 BCE), Lubrak (n=2; 1269-1123 BCE), Chokhopani (n=3; 801-770 BCE), Rhirhi (n=4; 805-767 BCE), Kyang (n=7; 695-206 BCE), Mebrak (n=9; 500 BCE-1 CE), and Samdzong (n=12; 450-650 CE). They are primarily chosen based on the availability but constitute a comprehensive time series including the earliest sites found in the region. We used each site as the analysis unit in most analyses, while combining all ancient MMD individuals in the selection scan analysis.
Sampling strategy	No sample-size selection was performed prior to the study. To produce ancient genomes reported in this study, we screened the accessible skeletal elements from the relevant geographic regions and time periods, and produced in-depth sequencing data for those with sufficient endogenous DNA preservation and without substantial contamination.
Data collection	For the screening of human DNA preservation, DNA extraction and library preparation of 50 skeletal samples were performed in the University of Oklahoma (OU) by Christina Warinner, Richard Hagan and Nisha Patel, and they were shallow sequenced at the University of Chicago on an Illumina HiSeq 4000 using 2x75 bp chemistry. Suila (n=2) and Lubrak (n=2) samples were extracted and built into libraries at MPI-SHH (by Raphaela Stahl) and screened on an Illumina HiSeq 4000 using 1x76 bp chemistry. Of these, 25 samples (21 individuals) were selected for a custom in-solution capture using oligonucleotide probes matching 50K manually selected target sites with functional significance at the University of Chicago (by Anna Gosling). All 47 well-preserved libraries were also captured for the 1240K panel SNPs at MPI-SHH (by Raphaela Stahl). 15 libraries were whole genome sequenced at Macrogen, Inc using an Illumina HiSeq X10 with 2x75 bp chemistry or MPI-SHH using an Illumina HiSeq4000 with 1x76 bp chemistry.
Timing and spatial scale	Laboratory works and sequencing were conducted over the period from March 2016 to December 2020. Samples were taken from seven high-altitude archaeological sites in the MMD region, Nepal. Detailed information of the archaeological samples studied in this manuscript is provided in Fig. 1 and Supplementary Data 1-4.
Data exclusions	We excluded samples only if the samples do not meet the quality criteria, either by having low level of endogenous human DNA prohibiting genome-scale sequencing or by showing high level of contamination estimates. For population genetic analysis that requires exclusion of genetic relatives, we excluded closely related individuals (1st degree relatives) by removing one with lower coverage from each pair.
Reproducibility	We took multiple individuals from each archaeological site, if available, to support the representativeness of their genetic profiles. For each sample, we estimated contamination level to support the authenticity of data. For nine samples (R2, KS20, KS25, KS23, KS21, KS8, MEB002, SZG003, SZG001; see Supplementary Data 1), we built both non-UDG and UDG-half double-stranded libraries and sequenced both libraries. Each of the nine library pairs produced genome-wide data confirmed to be from a single individual.
Randomization	Ancient genomes were first analyzed by each individual, and then were allocated into the analysis group based on their archaeological context and absolute date (14C dating). Randomization is not applicable.
Blinding	There was no experimental treatment of samples involved in this study that requires blinding. Data analysis was performed based on the analysis groups that were defined by external information (archaeological context and date).

Did the study involve field work? ☒ Yes ☐ No

Field work, collection and transport

Field conditions	The MMD sites are found within in a rain shadow of the Himalayas which has promoted relatively dry conditions and cool temperatures. For example, Mustang's capital Jomsom has an annual precipitation of 307 mm and mean annual temperature of 10.9 ˚C. Due to this, the preservation of organic and inorganic materials at archaeological sites in the region is excellent. Excavations were conducted at four cave complexes: Rhirhi, Sulia, Kyang, and Samdzong. A tomb exposed in a cutbank of a river, Lubrak, was also excavated. Two sites--Chokhopani and Mebrak--were excavated by other researchers and access to these materials from them was granted to Mark Aldenderfer and the project by the Department of Archaeology, Government of Nepal.
Location	The sites in this study are located in the Mustang and Manang districts of Nepal: Suila, 3900 masl, 28.98, 83.83; Lubrak, 3000 masl, 28.78, 83.84; Rhirhi, 3200 masl, 28.93, 83.82; Chokhopani, 2800 masl, 28.78, 83.72; Mebrak, 3600 masl, 28.82, 83.88; Kyang, 3900 masl, 28.31, 83.68; Samdzong, 4000 masl, 29.22, 84.03.
Access & import/export	All specimens reported in this manuscript were exported to co-author M.A. under the authority of the Department of Archaeology (DoA), Government of Nepal via the permits to the Sky Door Foundation Nepal/USA from 2007-to the present. Sampling of archaeological samples in the field was overseen by on-site representatives of the DoA at Rhirhi, Lubrak, Suila, Kyang, and Samdzong. Representatives of Village Development Committees (VDC) and other members of descendant communities were present during field sampling of archaeological materials at Rhirhi, Lubrak, Suila, and Samdzong. Those present were shown the material being taken for export and no objections were offered. Sampling of material from the Kapilvastu Museum was overseen by local staff and a representative of the DoA. Sampling of materials of the Mebrak remains was overseen at the DoA in Kathmandu. Approval for destructive analysis and genetic investigation was approved by the DoA as part of the permitting and export process.
Disturbance	All fieldwork was conducted as salvage excavation of sites already disturbed by earthquakes and other natural destructive factors.

Reporting for specific materials, systems and methods

We require information from authors about some types of materials, experimental systems and methods used in many studies. Here, indicate whether each material, system or method listed is relevant to your study. If you are not sure if a list item applies to your research, read the appropriate section before selecting a response.

Materials & experimental systems

n/a	Involved in the study	
☒	☐	Antibodies
☒	☐	Eukaryotic cell lines
☐	☒	Palaeontology and archaeology
☒	☐	Animals and other organisms
☒	☐	Human research participants
☒	☐	Clinical data
☒	☐	Dual use research of concern

Methods

n/a	Involved in the study	
☒	☐	ChIP-seq
☒	☐	Flow cytometry
☒	☐	MRI-based neuroimaging

Palaeontology and Archaeology

Specimen provenance	Sample numbers of all archaeological specimens are presented in Supplementary Data 1. All specimens were exported to co-author Mark Aldenderfer under the authority of the Department of Archaeology, Government of Nepal via the permits to the Sky Door Foundation Nepal/USA from 2007-to the present. Specific dates for export include: Aug/Sept 2010; Aug/Sept 2011; June/July 2012; Jan/Feb 2013; June/July 2014; Aug/Sept 2015.
Specimen deposition	All archaeological specimens not exported are currently in Nepal. Although the Nepali state technically has jurisdiction of all archaeological materials discovered within its borders, local Village Development Committiees take possession of them subject to negotiation with Department of Archaeology representatives in the field. The Chokhopani remains are in the Kapilvastu Museum in Tilaurakot; the Lubrak remains are located in the Tibetan Buddhist temple in the village of Lubrak; the Mebrak remains are housed in the Department of Archaeology facility in Kathmandu; the Kyang remains were reburied in the Kyang site in Manang; the Rhirhi materials are located in a small museum in the village of Chusang; the Suila materials are located in the village of Ghiling; and the Samdzong materials are housed in the community center of the village of Samdzong.
Dating methods	We provide 15 new accelerator mass spectrometry (AMS) dates from all seven archaeological sites included in this study. AMS dates for Suila, Lubrak, Rhirhi and Mebrak sites (n=6) are directly from the human skeletal elements used for the genomic analysis and are provided by UCIAMS (University of California, Irvine W.M. Keck Carbon Cycle AMS). AMS dates for Kyang and Samdzong sites (n=9) are from animal teeth or uncarbonized wood and are provided by MAMS (Curt-Engelhorn-Zentrum Archäometrie (Klaus-Tschira-Archäometrie- Zentrum), Mannheim, Germany). Detailed information is provided in the Supplementary Data 3. Calibration was performed using Calib 8.2 with IntCal20.

☒ Tick this box to confirm that the raw and calibrated dates are available in the paper or in Supplementary Information.

Ethics oversight	Sampling of archaeological samples in the field was overseen by on-site representatives of the Department of Archaeology, Government of Nepal at Rhirhi, Lubrak, Suila, Kyang, and Samdzong. Representatives of Village Development Committees (VDC) were present during field sampling of archaeological materials at Rhirhi, Lubrak, Suila, and Samdzong. Sampling of material from the Kapilvastu Museum was overseen by local staff and a representative of the Department of Archaeology, Government of Nepal. Sampling of materials of the Mebrak remains was overseen at the Department of Archaeology in Kathmandu. Approval for destructive analysis and genetic investigation was approved by the Government of Nepal, Department of Archaeology, as part of the permitting and export process (see above).

Note that full information on the approval of the study protocol must also be provided in the manuscript.
